# Proteome-based investigation of O-GlcNAcylation in a *C. elegans* model of ageing and Alzheimer’s disease: Functional support for earlier hypothesis-generating findings

**DOI:** 10.1371/journal.pone.0344865

**Published:** 2026-03-30

**Authors:** Fernando Garcia Olivera

**Affiliations:** 1 Department of Biochemistry, Faculty of Medicine, Justus-Liebig University of Giessen, Giessen, Germany; 2 Department of Biology, Faculty of Biology and Chemistry, Justus-Liebig University of Giessen, Giessen, Germany; Baylor College of Medicine, UNITED STATES OF AMERICA

## Abstract

O-linked N-acetylglucosamine (O-GlcNAc) is a post-translational modification of serine and threonine residues on nuclear and cytoplasmic proteins. The identity of O-GlcNAcylated proteins during ageing and neurodegenerative disease remains incompletely defined. Thus, animal models play a crucial role for the systematic characterization and cataloguing of O-GlcNAc-modified proteins. In this study, proteomic analysis was performed to identify O-GlcNAc-modified proteins in both L1 larval and adult stages of wild-type N2 and of *aex-3*p::Tau(V337M) transgenic *Caenorhabditis elegans*, a nematode model of ageing and Alzheimer’s disease (AD) using high-resolution nano-LC–ESI mass spectrometry. O-GlcNAcylated proteins identified in the N2 strain were mapped to nuclear- and RNA-related processes in both stages. In the tau-expressing strain, functional enrichment analysis of the identified proteins indicated a predominance of stress-response–related pathways. Together, these data present an analysis of O-GlcNAc-modified proteins across early development, adulthood, and a tau-related *C. elegans* model, providing a resource for future functional and comparative studies of O-GlcNAcylation in ageing and AD.

## Introduction

According to the World Health Organization, Alzheimer’s disease (AD) is the most prevalent type of dementia, and although age is the strongest known risk factor for its onset, AD is not an inevitable consequence of ageing. Furthermore, dementia does not only afflict the elderly, up to 9% of dementia cases are classified as young-onset, defined by symptom onset before the age of 65 [1]. However, the molecular mechanisms that mediate the transition from healthy ageing to pathological decline remain incompletely understood.

Among the emerging modulators of neuronal function and proteostasis is O-linked β-N-acetylglucosaminylation (O-GlcNAcylation), a dynamic and reversible post-translational modification (PTM) that regulates thousands of proteins involved in metabolism, stress response, transcription, and proteostasis. Acting as a cellular metabolic sensor, O-GlcNAcylation is tightly coupled to glucose metabolism via the hexosamine biosynthetic pathway [2,3]. In the ageing brain, decreased O-GlcNAcylation has been associated with increased tau hyperphosphorylation and enhanced amyloidogenic processing, both of which are central to AD pathology [4,5].

O-GlcNAcylation is a post-translational modification involving the addition of a single GlcNAc moiety to serine or threonine residues. It is dynamically regulated by O-GlcNAc transferase (OGT) and O-GlcNAcase (OGA), and although it can occur co-translationally, it is generally considered a post-translational event [6]. In *Caenorhabditis elegans*, the OGT-1 and OGA-1 enzymes are highly conserved and functionally comparable to their human counterparts. They are essential for maintaining cytoplasmic and nuclear proteostasis. Previous studies have shown that mutations in these enzymes modulate Aβ (Beta-amyloid) and tau toxicity in *C. elegans* models, supporting the use of this model organism to explore the molecular roles of O-GlcNAc in ageing and neurodegeneration, as well as to identify small-molecule modulators of O-GlcNAc cycling [7,8].

Although studies have reported inconsistent findings regarding global protein O-GlcNAcylation levels in AD, there is growing recognition that O-GlcNAcylation is dysregulated in the disease [9]. However, there is no clear consensus on the specific nature of these alterations, as different studies report divergent results. Some suggest a general decrease in O-GlcNAcylation, while others indicate that changes are more selective or region-specific. Moreover, the interplay between O-GlcNAcylation and other glycosylation pathways may contribute to the complexity of these modifications in AD. For instance, Wang et al. and Frenkel-Pinter et al. reported significant differences in specific O-GlcNAc-modified peptides in AD brains compared to controls, suggesting that some proteins may undergo reduced modification, whereas others may be upregulated or modified in distinct ways [10,9].

While O-GlcNAcylation of other proteins, including the amyloid precursor protein (APP), has been investigated in the context of AD, tau remains a central target due to its critical role in tangle formation. Most of the research has focused on aberrant tau O-GlcNAcylation as a driver of tau pathology in AD, reinforcing the hypothesis that this PTM is a crucial modulator of tau function and aggregation [11]. Nevertheless, given the broad scope of O-GlcNAcylation across many proteins, its dysregulation may affect multiple cellular pathways involved in AD pathogenesis beyond tau.

In this study, we used a proteomic approach, employing the wild type (WT) N2 strain (Bristol) and a transgenic strain of *C. elegans* expressing the human 4R1N tau isoform with the AD-linked V337M mutation under the control of the pan-neuronal *aex-3* promoter, to systematically identify O-GlcNAcylated proteins at the L1 post-embryonic larval and adult stages. Furthermore, functional enrichment and protein-protein interaction analyses of the O-GlcNAc-modified proteins showed distinct enrichment patterns. RNA-related processes were enriched in the WT N2 strain at both L1 and adult stages, whereas stress-responsive pathways were enriched in the tau transgenic strain. Collectively, these findings provide identified datasets of O-GlcNAcylated proteins across stages and genotypes of *C.elegans* that may be used to support future quantitative and mechanistic studies of O-GlcNAcylation in ageing and tauopathy-related models.

## Materials and methods

### Worms synchronization

The WT N2 strain (Bristol) and the *aex-3*p::Tau(V337M) transgenic strain (University of Washington, Seattle, USA) were used for our experiments. The *aex-3* promoter drives the expression of the human tau V337M transgene predominantly in neurons and is commonly used in *C. elegans* for pan-neuronal expression, making it a suitable genetic tool to model tauopathy and investigate neuron-specific effects of post-translational modifications [12]. A solid sterile Nematode Growth Medium (NGM) was prepared by dissolving 17 g agar (cat. no. 8503), 3 g NaCl (cat. no. S7653), 2.5 g peptone (cat. no. 91079-38-8) in distilled water to a final volume of 1 L. After autoclaving, the medium was supplemented with 1 ml 1M CaCl_2_ (cat. no. 102378); 5 mg/ml cholesterol (cat. no. C8503), 1 ml 1 M MgSO_4_ (cat. no. 0261.1), 25 ml 1 M KPO_4_ buffer (cat. no. 3904.1 and P290), poured into 94 x 16 mm plates (Greiner Bio-One, Frickenhausen, Germany) and fed with *Escherichia Coli* OP50 (Protein analytics, Giessen (HE), Germany) as food source to propagate *C. elegans* strains according to standard procedures [13]. All strains were grown at a temperature of 20°C. These plates were grown for 24 h in an incubator (MAGV, Rabenau, Germany). Every two to three days, they were moved to a plate containing a new NGM medium. Six plates of adult worms of each strain, to obtain 30.000 eggs, were bleached using the alkaline hypochlorite method to prepare synchronized animals, respectively [13]. In a nutshell, worms were removed from culture plates using M9 buffer composed of 22 mM KH_2_PO_4_ (cat. no. P290), 42 mM Na_2_HPO_4_ (cat. no. S7907), 86 mM NaCl (cat. no. S7653) and 1 mM MgSO_4_ (cat. no. 0261.1). After removing all the bacteria from the supernatant, they were lysed in a solution of alkaline hypochlorite (NaOCl, cat. no. 9062−3) for 1 minute and neutralized by using M9 medium; this process was monitored under a dissecting microscope and repeated six times until worms broke apart. Debris was washed with M9 by centrifugation at 2000 rpm for 3 min at 4°C (Hettich® Universal 32R Centrifuge, Tuttlingen, Germany) until the solution was cleared (eight wash cycles). The obtained eggs were then cleaned by centrifugation at 1800 rpm for 3 min using M9 buffer at 4°C and allowed to nutate overnight before hatching in 2 ml of M9 in a climatic chamber (Liebherr®, Ochsenhausen, Germany) at 20°C.

### Worm lysis and protein extraction

Aliquots of worm eggs of WT N2 and of *aex-3*p::Tau(V337M) strain were pipetted in 15 ml tubes containing 1 ml M9 medium. These were let mature in the mentioned solution overnight. Aliquots of L1 hatchlings were seeded onto brand-new NGM plates containing OP50 culture. this population was grown at 20°C for 10 days. Another L1 *C. elegans* larvae from the corresponding WT N2 and the transgenic strain were rinsed three times and kept for protein extraction. On day 11, worms were collected by adding 3 ml of M9 buffer to each plate. The plates were gently swirled and the solution was filtered through a 50 μm mesh (Cytecs, Muenster, Germany) and thoroughly rinsed with M9 buffer. This washing procedure was performed twice to obtain a population of adult stages. The nematodes that remained on the 50 μm mesh were carefully transferred into a 15 ml tube and centrifuged for three minutes at 1800 rpm at 4 °C. The supernatant was removed and the worms were resuspended with 10 ml of M9 buffer and centrifuged three times, discarding the supernatants. The washed L1-larvae and the retained fraction of adult nematodes were then transferred to 1.5-ml microfuge tubes separately and concentrated by centrifugation (1800 rpm, 3 min, 4 °C), the supernatant was removed, and 300 μl of ice-cold lysis buffer was added (150 mM NaCl, cat. no. S7653; 20 mM Tris, cat. no. 4855.2 pH 7.4; 1.0% Triton X-100, cat. no. 9002-93-1) supplemented with protease inhibitor cocktail (cat. no. 04693132001). The suspended worms were pelletized again at 1800 x rpm for 3 min and the tube was frozen in liquid nitrogen, followed immediately by rapid thawing in a 37°C water-bath and disrupted by subjecting the samples to vortex pulse cycles of 15 s each (Vortexer, Heidolph, Schwabach, Germany). This freeze-thaw cycle was repeated three times, cooling on ice between pulses. After the second freeze-thaw cycle, the lysis of samples was enhanced by using two ultrasound pulse cycles of 15 s (Ultrasonic bath, Elmasonic S40, Singen, Germany). Lysates were maintained on ice and examined using a light microscope to verify successful lysis of the worm.

The sonicated worm lysates were centrifuged at 14,000 g for 10 min at 4°C to pellet any worm-debris. Subsequently, the lysates supernatants were transferred to a 15 ml conical tube at 20°C–25°C. Worm lysates were made up the total volume to 1.8 ml. Total protein amount was measured in the range of 1–4 mg per tube.

### Protein precipitation, resuspension, and quantification

3 volumes (6 ml) of ice-cold acetone were added to the worm lysates to precipitate proteins and mixed well by inverting a few times. The samples were incubated overnight at −20°C to aid precipitation. Precipitated proteins were pelleted by centrifugation at 4,000 g for 30 min at 4°C. The supernatants were carefully decanted without disturbing the pellets. Any remaining acetone was removed and then the pellet was air-dried for 15 min by leaving the uncapped tube at 20°C–25°C.

Pellets were resuspended in 200 μl of a buffer containing 100 mM NH_4_HCO_3_, by pipetting up and down. Protein quantification was performed using the Pierce BCA protein assay kit (cat. no. A55864) and measured with an absorbance microplate reader (BioTek ELx800TM, Washington, USA) according to the manufacturer instructions. The protein lysates were reduced for 30 min at 37°C with 5 mM DDT (cat. no. D0632) and subsequently alkylated using 15 mM iodoacetamide (cat. no. 144-48-9) for 30 min at room temperature in the dark and digested overnight at room temperature with sequencing grade trypsin (MS gold-grade, Mannheim, Germany) at an enzyme ratio of 1:50 (w/w) to a final reaction volume of 500 μl. the digestion of samples was stopped with trifluoroacetic acid (TFA, cat. no. 302031) at a final concentration of 0.1%.

To remove any remaining insoluble material that had not been digested, samples were centrifuged at 14,000 g for 20 min at room temperature. The supernatant, which included the peptide mixture, was then collected in Lobind® tubes (Eppendorf cat. no. 56251, Hamburg Germany). The remaining insoluble peptide particles were extracted by adding 0.5 ml of 0.1% TFA in water, pipetting the pellets back into suspension and then centrifuging them once more for 20 min. The supernatant was combined with the prior one, desalted by using strong C18 solid-phase extraction cartridges (Macherey-Nagel cat. no. 730011, Dueren, Germany). Detergent traces were removed by incubation and centrifugation using Pierce^TM^ removal spin columns (Thermo Fisher Scientific cat. no. 87777, Dreieich, Germany) and dried completely using SpeedVac (Eppendorf, Concentrator Plus^TM^, Hamburg, Germany).

### Peptide enrichment

We used a label-free alternative to enrich and detect O-GlcNAc-modified proteins, by employing s-*WGA* (*succinylated wheat germ agglutinin*), a lectin that binds specifically to terminal N-acetylglucosamine (GlcNAc) residues. Succinylation of *WGA* reduces its affinity for sialic acid and other non-specific glycan structures, thereby increasing its selectivity towards β-linked GlcNAc residues typical of O-GlcNAc modifications on intracellular proteins [14,15]]. Unlike native *WGA*, which exhibits broader carbohydrate-binding activity, s-*WGA* offers improved specificity for cytoplasmic and nuclear O-GlcNAcylation without substantial cross-reactivity to complex N-glycans or membrane-associated glycoconjugates [16,17]. This increased specificity makes s-*WGA* particularly suitable for applications such as lectin blotting, affinity purification, and gel-based mobility shift assays targeting O-GlcNAc-modified proteins [18,19,20]. Thus, s*WGA* provides a reliable and widely accepted tool for detecting O-GlcNAc in diverse biological samples, especially when antibody-based approaches may lack sensitivity or cross-react with other glycans.

For our study, 1 mg of dried peptide pellet were dissolved in 1 ml of a 50 mM sodium phosphate buffer, 7.0, containing 20 mM NaCl (Binding buffer, cat. no. 21511271−1) and then incubated overnight at 4°C with a 200 µL slurry containing s-*WGA* – MagneZoom™ beads (cat. no. 20120079−1; Dublin Ohio, USA) using rotational mixing (IKA Loopster basic, Staufen, Germany). The beads were precipitated by centrifugation at 1000 g for 5 min and washed by adding 0.5 ml of binding/wash buffer and centrifuging again to remove the supernatant, the washing process was repeated two additional times.

Tubes containing samples were removed from the centrifuge. Bound peptides were eluted by adding 300 μl of 50 mM Sodium Phosphate buffer containing 0.1–0.2 M N-acetylglucosamine and 500 mM NaCl (Elution buffer, cat. no. 21511313−1). The beads were resuspended and incubated at room temperature for 15 min with rotational mixing. Following incubation, the beads were centrifuged at 1000 g and the supernatant was transferred to a clean tube. This incubation and elution were repeated three times using elution buffer. The flow through was then collected and desalted using strong solid-phase C18 cartridges (Chromabond) and dried using a SpeedVac concentrator for the LC-MS/MS analysis.

### Nano-LC-MS/MS analysis and evaluation of O-glycan

1 μg from the glycopeptides enriched above were redissolved in 0.1% formic acid (cat. no. 27001) and loaded onto a 50 cm µPAC™ C18 column (Pharma Fluidics, Gent, Belgium). The peptides were eluted using a linear gradient from 9 to 50% of 20 mM NH_4_OCH_3_, pH 10 in 90% acetonitrile over 240 min at a constant flow rate of 300 nL/min (Thermo Fisher Scientific™ UltiMate™ 3000 RSLC nano), infused via an Advion TriVersa NanoMate (Advion BioSciences, Inc. New York, USA) and analysed by an Orbitrap Eclipse Tribrid mass spectrometer (Thermo Fisher Scientific, Waltham, MA, USA. Each precursor ion (charged 2–7+) was separated in the quadrupole (selection window: 1.6 m/z; dynamic exclusion window set to 30s and a 3s scan cycle by using EThcD fragmentation (Maximum Injection Time: 250ms; normalized Collision Energy: 25%) and a resolution of 30,000; AGC; 5.0e4) to trigger HexNAc oxonium ion fragments.

Raw mass-spectra data files were processed for intact O-glycopeptides identification using Mascot Distiller (Matrix Science, London, UK, v2.8.5.1 version), a software that processes high-quality peak lists for Mascot database searches. The following parameters were set; Species, *C. elegans*; enzyme specificities (Trypsin, Digest C Term (KR) with a maximum of two missed cleavages; fixed modification, carbamidomethylation of Cys (C); variable modifications were HexNAc (ST) and Methionine oxidation (M). The mass accuracy was 0.08 Da for-precursor ions and 0.05 Da for fragment ions. Annotation results were filtered to obtain peptide spectrum matches (PSMs) with a ≤1% FDR (False Discovery Rate). The search of corresponding O-glycopeptides was conducted against the current secretome UniProtKB/Swiss-Prot database, contaminants, and UP1940 for *C. elegans*.

Peptides among this range were considered O-GlcNAcylated candidates and were manually inspected by diagnostic of HexNAc oxonium ions (m/z 204.0867 with 138.055 and/or 144.066), observation of a 203.0794 Da neutral loss, and sequence support from b/y ions of the modified residue. Expectation Value (E-Value) less than 0.05, Mascot ion score more than 20, and <30 were set as acceptance criteria. HexNAc on S/T with an ion score ≥30, was considered as strong evidence of O-GlcNAc glycopeptide after a positive manual inspection with a mass error between 5–10 parts per million (ppm) and scores of 40 or higher were designated very high-confidence [21,22]. Violin plots representation of distribution between parameters for all the identified sequences was performed using OriginPro 2025 (Origin Lab Corporation).

### Identification of orthologs

Orthologs protein identification was performed using the UniProt database in Homo sapiens sequence identity of 100% and OrthoList2, a database that compiles *C. elegans* and human orthologs based on a 2018 meta-analysis. (Table in S1 Table), according to conservative standards commonly applied in recent large-scale studies of protein orthology and comparative genomics [23,24].

### Protein-Protein Interaction network (PPI) analysis

A protein-protein interaction network analysis was performed using the STRING v12.0 database to identify protein associations and functional enrichment among proteins identified with at least one O-GlcNAc-modified peptide. STRING analysis were assessed according to the maximum possible number of edges between neighbours in a fully connected graph of equivalent size and structure [25]. For the WT N2 strain, a maximum of 21 interactions per protein were permitted. For proteins detected in *aex-3*p::Tau(V337M), only one first-shell interaction per protein was allowed. To further explore network connectivity, the network was subsequently expanded by adding 20 second-shell interactors while maintaining a total of 21 interactions per protein. A high confidence interaction score threshold of 0.7 was applied. Protein-protein interactions were inferred from the full STRING network using evidence derived from text mining, experimental data, genomic neighbourhood, phylogenetic co-occurrence, co-expression, and curated public [26]. Over-represented functional terms were filtered based on STRING-reported, multiple-testing-adjusted false discovery rate (FDR) values (FDR: < 0.01, FDR: < 0.001, or FDR: < 1 $\cdot 10^{-4}$). Reported enrichments are conditional on interaction identification and are intended to support hypothesis generation.

## Results

### Global identification of O-GlcNacylated proteins

Proteomic analysis was performed to identify O-GlcNAcylated protein targets in nematodes across ageing and during the progression of AD pathology. The characterization phase initiated by using collected samples of WT N2 and *aex-3*p::Tau(V337M) *C. elegans* to search for protein sequences containing motifs corresponding to O-GlcNAcylation. O-GlcNAcylated peptides annotated by Mascot were subjected to additional layers of validation to ensure confident assignment. Only PSMs with scores exceeding the identity threshold (expect: < 0.05) were considered further. Inspection of the corresponding MS/MS spectra revealed diagnostic HexNAc oxonium ions at serine/threonine residues (m/z 204.0867 and related fragments), as well as neutral-loss signals (−203Da) characteristic of O-GlcNAc. Oxidation (M) was also reported on one peptide as variable PTM. Backbone fragmentation was assessed to determine coverage of the modified residue. The analysis included both intact b and y ions as well as neutral-loss derivatives, specifically y* ions with loss of ammonia ($-NH_{3}$) and y ions with loss of water ($-H_{2}O$). Only ions with a mass error of 5–10 ppm or less were considered. Both singly and doubly charged states were evaluated using extracted ion annotations for each strain (please refer to S1 Dataset and Table in S1 Table). Peptides with Mascot ion scores greater than 20 and <30 were considered candidate O-GlcNAcylated peptides. Scores above 30 indicated high confidence and scores of 40 or higher represented excellent confidence.

### High-confidence peptides (score ≥ 30)

Most annotated O-glycopeptides achieved scores above the significance threshold, ensuring reliable identification. Representative examples included: PNSRHDNVSPSK (score 41.0; Mr(calc): 1742.81), identified with HexNAc modifications at S9 and S11. LLVADILACNDDTPASAMMAGNGPVATMSLQVK (score 43.2; Mr(calc): 3519.70), containing a glycosylation at S29. HGGTTRTADAIRYATK (score 33.6; Mr(calc): 2124.04), observed in multiple HexNAc-modified variants in WT N2 adult and in *aex-3*p::Tau(V337M) L1. These high-scoring peptides demonstrated extensive fragment ion coverage and reproducible detection across replicate (n = 2) runs. Collectively, they provided confident assignments to proteins such as glutamic acid-rich protein, K homology protein domain, and vWFA (von Willerbrand factor type A)-domain containing protein.

### Candidate peptides (score <30)

**Fig 1:**
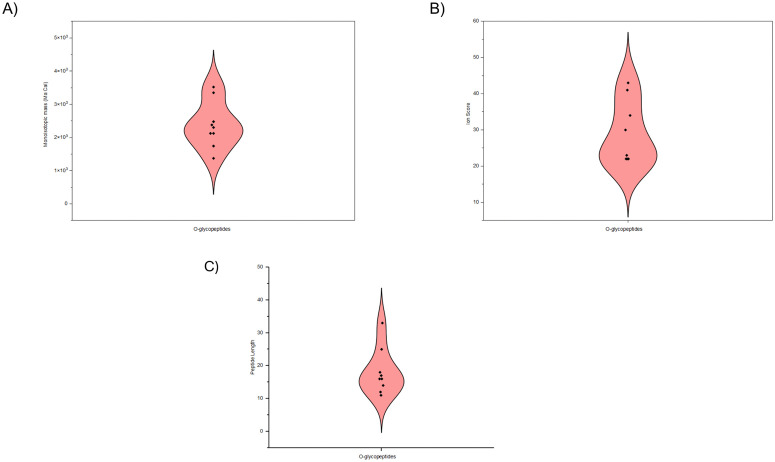
Violin plots representation of distribution between parameters for all the identified O-glycopeptide sequences. A. Monoisotopic molecular weight (Ma Cal). B. Ion score. C. P**e**ptide length. Each plot displays the distribution density and individual peptide values.

A smaller subset of annotated peptides did not reach the Mascot confidence threshold; however, these O-glycopeptides were classified as reliable identifications based on consistent tandem mass spectrometry (MS/MS) fragmentation patterns. These included: VGLIAARRTGR (score 22; Mr(calc): 1371.79), mapping to ribosomal protein uL2 (UniProt: *RL*8_*CAEEL*). RARDSASSSSSHSK (score 23.0; Mr(calc): 2477.10), derived from a spliceosome-associated protein (UniProt: *CWC*15_*CAEEL*). DELPAIRLISLEEDMTK (score 22.2; Mr(calc): 2378.18), exhibiting HexNAc ions on both Ser and Thr. MFITRGLILISLLFVFVMTDDTHDK (score 22.1; Mr(calc): 3346.71), heavily modified with HexNAc and oxidation. TFDFRADKILESLTNSLK (score 22.2; Mr(calc): 2300.19), derived from a NADP-dependent oxidoreductase domain- containing protein. While these sequences did not surpass the significance threshold, their pExpect values were below 0.05. Such candidates require further validation using targeted MS/MS or synthetic peptide standards. The Table in S1 Table and the data in S2 Dataset present a comprehensive summary of all the annotated Mascot sequences. Collectively, peptide data from the WT N2 and *aex-3*p::Tau(V337M) samples were processed and evaluated to obtain identification profiles. The monoisotopic mass distribution of identified O-glycopeptides exhibited an asymmetric pattern, with pronounced concentration in medium and high-mass range (Fig 1A). Ion scores indicated confidence levels that are generally regarded as acceptable for reliable spectral matching in proteomic identifications. The asymmetrical ion score distribution suggests variability in peptide identification confidence across the datasets (Fig 1B). In addition, most O-glycopeptides were distributed between the length of 15–19, suggesting a predominance of moderately sized peptides (Fig 1C).

### Integration of O-GlcNAcylation within protein networks and pathways in ageing and AD model

**Fig 2:**
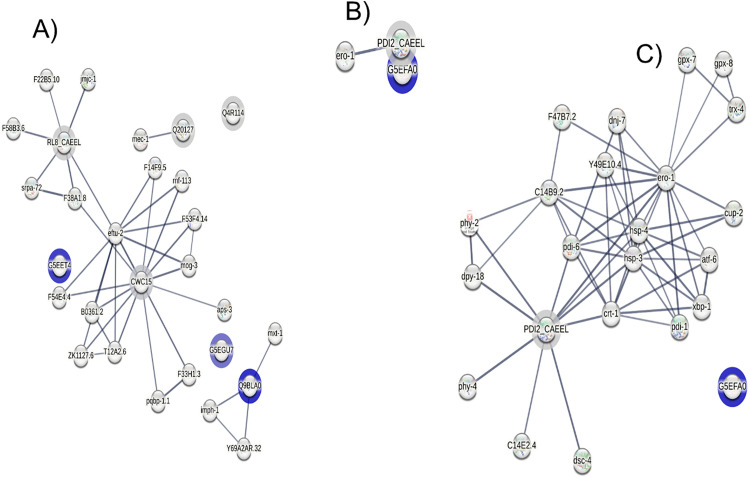
Functional enrichment of identified O-GlcNAcylated proteins identified in a *C. elegans* model of ageing and AD. **A)** STRING-based PPI analysis of O-GlcNAcylated proteins identified in L1 stage and adult worms of the WT N2 strain (based on 21 first-shell interactions). **B)** STRING-based PPI analysis of O-GlcNAcylated proteins identified in L1 stage, and adult worms of the *aex-3*p::Tau(V337M) strain (based on one first-shell interaction). **C)** STRING-based PPI analysis of O-GlcNAcylated proteins identified in L1 stage, and adult worms of the *aex-3*p::Tau(V337M) strain (based on a second-shell expansion for a total of 21 1st- and 2nd-shell interactions). Proteins identified with a blue halo represent those with high and very high confidence (ion score ≥30 and pExpect: < 0.05).

**Fig 3:**
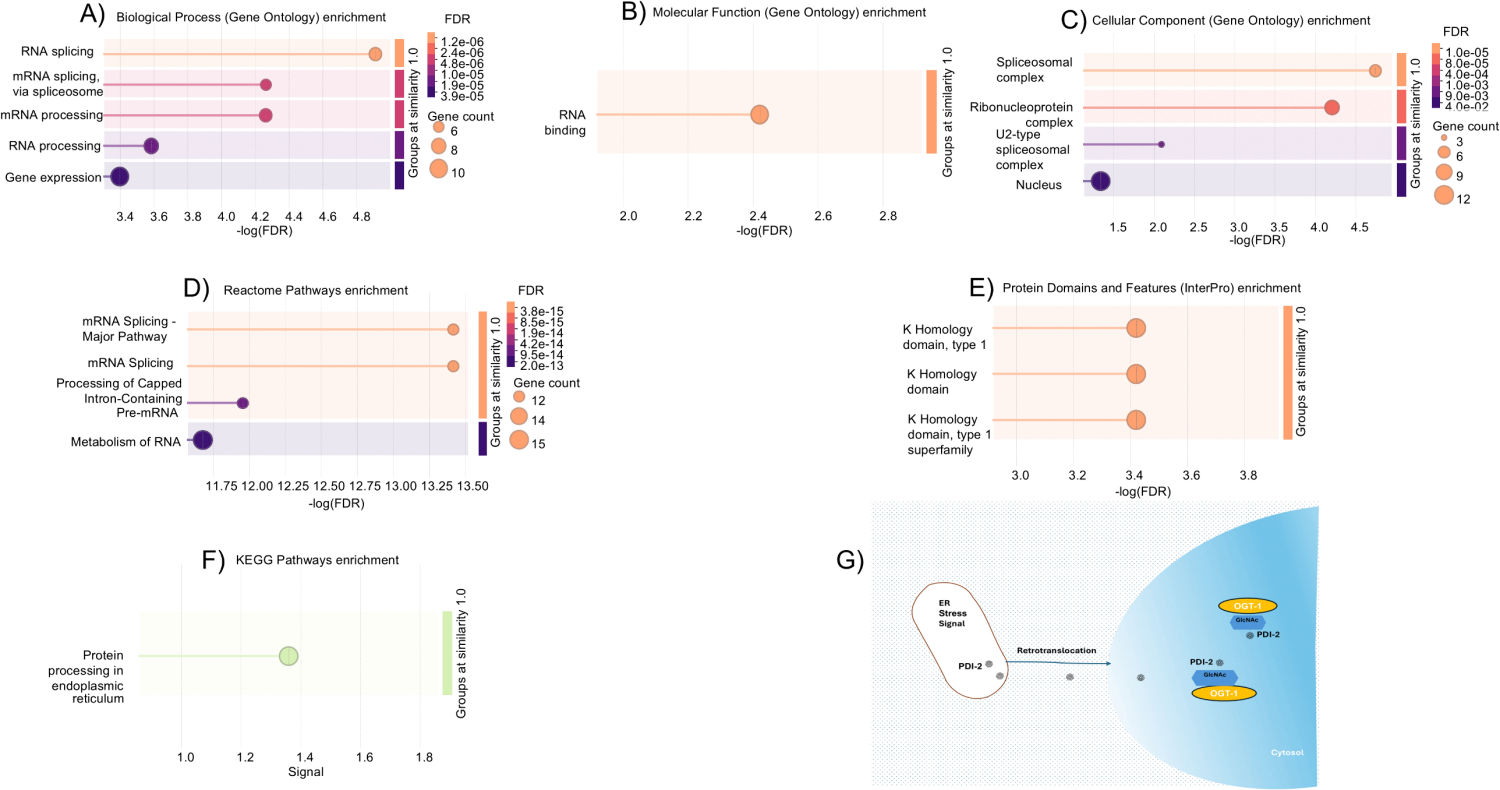
Bubble charts of enrichment terms and hypothetical schematic of non-canonical protein O-GlcNAcylation of an ER-resident protein. A Biological process. **B)** Molecular function. **C)** Cellular component. **D)** Reactome Pathways. **E)** Protein Domains and features enrichment in the WT N2 strain. **F)** KEGG Pathways enrichment in the *aex-3* transgenic strain). **G)** Non-canonical O-GlcNAcylation: Relocated PDI-2 (UniProt: *PDI*2_*CAEEL*) modified in cytosol).

Functional enrichment analysis of O-GlcNAcylated proteins in the N2 strain was conducted using the STRING PPI network (Fig 2A). In the biological process (BP) category, gene ontology (GO) annotations indicated enrichment in RNA splicing (GO:0008380, FDR: < 1 $\cdot 10^{-4}$), mRNA splicing, via spliceosome (GO:0000398, FDR: < 1 $\cdot 10^{-4}$), mRNA processing (GO:0006397, FDR: < 1 $\cdot 10^{-4}$) as shown in Fig 3A and Table A in S3 Dataset. The molecular function (MF) category was enriched for RNA binding (GO:0003723, FDR: < 0.01) (Fig 3B and Table B in S3 Dataset). For the cellular components (CC) category, the enriched genes were primarily associated with spliceosomal complex (GO:0005681, FDR: < 1 $\cdot 10^{-4}$) as well as ribonucleoprotein complex (GO:1990904, FDR: < 1 $\cdot 10^{-4}$) (Fig 3C and Table C in S3 Dataset). Reactome annotations indicated involvement in mRNA splicing-Major Pathway, mRNA splicing, processing of capped intron-containing Pre-mRNA and metabolism of RNA (FDR: < 1 $\cdot 10^{-4}$) (Fig 3D and Table D in S3 Dataset). Interestingly, InterPro database enrichment analysis identified an overrepresentation of K homology (KH) domains in the dataset, including the KH domain, KH domain type 1, and KH domain superfamily (FDR: < 0.001) (Fig 3E and Table E in S3 Dataset).

In parallel, a PPI network analysis was performed for O-GlcNAcylated proteins detected in *aex-3*p::Tau(V337M) strain (Fig 2B). While the first shell included only one direct association among the query proteins with an enrichment in protein processing within the endoplasmic reticulum (ER) (Kyoto Encyclopedia of Genes and Genomes (KEGG) pathway, cel04141; FDR: < 1 $\cdot 10^{-4}$) (Fig 3F and Table A in S4 Dataset), expansion to the second shell substantially increased the number of associations by incorporating proteins directly connected to the original set (Fig 2C). BP terms suggests an over-represented enrichment for processes, including ER unfolded protein response (UPR), response to ER stress and IRE1-mediated UPR (GO:0030968, GO:0034976 and GO:0036498; FDR: < 1 $\cdot 10^{-4}$) (Fig 1A in S1 Fig and Table B in S4 Dataset). Second-shell expansion introduced MF terms associated with protein disulfide isomerase activity, oxidoreductase activity and procollagen-proline 4-dioxygenase activity (GO:0003756, GO:0016491 and GO:0004656; FDR: < 1 $\cdot 10^{-4}$) (Fig 1B in S1 Fig and Table C in S4 Dataset). CC terms mapped predominantly to the ER lumen, ER, endomembrane system and cytoplasm compartments (FDR: < 1 $\cdot 10^{-4}$) (Fig 1C in S1 Fig and Table D in S4 Dataset) (Fig 1C in S1 Fig and Table D in S4 Dataset). Reactome pathways analysis indicated over-representation of post-translational protein phosphorylation, regulation of Insulin-like Growth Factor (IGF) transport and uptake by Insulin-like Growth Factor Binding Proteins (IGFBPs), and post-translational protein modification (FDR: < 1 $\cdot 10^{-4}$) (Fig 1D in S1 Fig and Table E in S4 Dataset). The expanded PPI further revealed significant enrichment of annotations from the InterPro database corresponding to the thioredoxin domain and the thioredoxin-like superfamily (FDR: < 1 $\cdot 10^{-4}$) (Fig 1E in S1 Fig and Table F in S4 Dataset).

To investigate conservative homology associated with ageing and AD pathology in humans, we performed a query of *C. elegans* O-GlcNAcylated proteins against Ortholist 2 [24] and UniProt Database, a percentage of 78% of the queried genes were identified to have human orthologs (Table in S1 Table). Comparison of ortholog identification indicated that seven genes were annotated in UniProt as having human orthologs, whereas OrthoList2 (Table in S2 Table) identified only five. Additionally, two additional genes were found to lack orthologs in both databases. This discrepancy reflects differences in database coverage and curation criteria. Nonetheless, this integrative approach highlights evolutionarily conserved molecular targets between humans and the *C. elegans* AD model, providing a basis for further interpretation in the context of ageing and AD mechanisms.

## Discussion

This proteomic study identified stage and strain-specific O-GlcNAcylated proteins in *C. elegans*, encompassing L1 post-embryonic larval and adult stages in the WT N2 strain and in the *aex-3* transgenic background. Integration of high-resolution mass spectrometry data with protein-protein interaction (PPI) network analysis enabled organization of the identified O-GlcNAcylated proteins into recurring enrichment terms, including RNA-related, and stress-associated processes. These analyses provide a systems-level description of the distribution of O-GlcNAcylated proteins within the detected proteome.

The protein set in the WT N2 strain showed overrepresentation of nuclear ribonucleoprotein assemblies, with spliceosomal complex terms among the most significantly enriched GO annotations. Consistent with this, most identified O-GlcNAcylated proteins were assigned to the nucleus, indicating preferential recovery of nuclear, RNA-related components in the WT strain N2 protein set. Integrative analysis of the PPI network and pathway enrichment data suggests that the set of O-GlcNAcylated proteins occupies a highly interconnected region of the cellular interaction network in WT N2 L1 strain. Within this network, central nodes included the CWC15 homolog and the ribosomal protein RL8, both associated with RNA binding and roles in RNA metabolism processes. The presence of these proteins within the same interaction clusters indicates co-occurrence between putative O-GlcNAcylated proteins and components assigned to RNA-related processes at early larval stage. Additional O-GlcNAcylated proteins detected in WT larvae (N2 L1) that did not map to these clusters was Q20127, associated with mechanosensory perception and oxidoreductase activity. Other identified proteins included glutamic acid-rich protein (UniProt: G5EET4) and PIR protein (UniProt: Q4R114). Although these proteins have been linked to signalling or structural processes according to existing UniProt database annotations, their precise functions during this developmental stage remain undetermined. An O-GlcNAcylated peptide carrying an additional, variably assigned PTM was identified, which may reflect the coexistence of multiple modifications within peptides from the WT N2 L1 strain. In particular, the peptide sequence MFITRGLILISLLFVFVMTDDTHDK annotated to a PIR protein (UniProt: Q4R114), was detected with a concurrent methionine oxidation (M18*y2 + , neutral loss of m/z 63.99). The presence of this oxidative modification is consistent with previously reported redox-sensitive PTM sites that have been associated with protein folding or glycosylation efficiency [27]. While this study is primarily focused on PTM identification, the observed co-occurrence of O-GlcNAcylation and oxidation is in agreement with prior reports, including Fahie et al. [28], suggesting a potential intersection between O-GlcNAc signalling and oxidative stress-related pathways under nutrient-limited conditions.

In adult WT N2, O-GlcNAc-modified protein subset included the KH domain-containing protein, isoform b (Q9BLA0) with GO annotations related to RNA binding, mRNA-processing and mapped to RNA metabolism; and a vWFA domain-containing protein (UniProt: G5EGU7), associated with collagen-related processes and cell surface interactions according to reactome annotations from UniProt. Although network topology analysis showed that these proteins have reduced connectivity to ribosomal and spliceosomal modules, InterPro enrichment analysis indicates an overrepresentation of KH domains, which have been previously reported as RNA-binding modules associated with RNA splicing, stability, transport, and translation [29,30]. A reported O-GlcNAc modification of FUBP1, the human ortholog of *Q*9*BLA*0_*CAEEL*, raises the possibility that KH domain-containing proteins could be conserved targets of this PTM [31].

In the *aex-3*p::Tau(V337M) AD model, O-GlcNAcylation of a vWFA domain-containing protein (UniProt: G5EFA0) was detected in the L1 stage. Based on gene annotations from UniProt, this protein is associated with extracellular matrix-associated interactions. The presence of this modification is noted in the context of the AD tauopathy model; however, its functional significance, including any relationship to neuronal signalling remains to be determined and will require a functional-targeted analysis. PDI-2 (UniProt: *PDI*2_*CAEEL*) was identified as a putative O-GlcNAcylated protein in the *aex-3*p::Tau(V337M) adult worms. Members of the protein disulfide isomerase (PDI) family contain thioredoxin domains, consistent with the enrichment of stress-response processes identified by the PPI analysis of the expanded network. While the observed enrichment terms from the expanded network likely reflect shared interaction contexts rather than direct functional roles of the original seed proteins, post-translational modification of PDIs by stress-responsive pathways has been increasingly reported in the context of neurodegenerative diseases. In light of reports describing ER protein reflux into the cytosol under proteotoxic stress conditions [32,33], PDI-2 may be subject to post-translational modification outside the ER, potentially including O-GlcNAcylation (Fig 3G). In line with this possibility, human studies have reported colocalization of PDI proteins with cytoplasmic TDP-43 aggregates in amyotrophic lateral sclerosis (ALS) and AD, suggesting a conserved association with stress responses [34,35].

Although *C. elegans aex-3*p::Tau(V337M) strain provides a robust model for tau-driven pathology, several limitations must be considered. Transgenic lines exhibit reduced lifespan and progeny viability compared to WT N2, complicating population synchronization and long-term experiments [36]. This proteomic approach achieved broad coverage; however, O-GlcNAcylated candidates with lower ion scores require cautious interpretation and should be validated using targeted analytical methods. Cross-species extrapolation is limited by species-specific differences in O-GlcNAc cycling and neuronal physiology, despite the identification of conserved orthologs. Further investigation is necessary to clarify the causal relationships between O-GlcNAcylation and other post-translational modifications, such as phosphorylation and oxidation.

## Conclusion

In summary, this study presents a proteomic analysis focused on identifying O-GlcNAcylated proteins in L1 larval and adult stages across different genetic backgrounds in *C. elegans*. High-resolution mass spectrometry, combined with protein-protein network-based enrichment and functional enrichment analyses, showed stage- and strain-specific O-GlcNAcylation patterns. O-GlcNAcylated proteins in N2 L1 larvae were predominantly enriched in nuclear and RNA-related processes, including spliceosomal and ribonucleoprotein complexes. RNA-binding-related processes were enriched among identified proteins in both L1 and adult stages, with a KH domain-containing protein isoform detected specifically in adult worms. In the tau(V337M) transgenic strain, putative O-GlcNAc-modified PDI-2 was significantly enriched in pathways related to stress-responsive processes, including the unfolded protein response. In line with prior observations of distinct O-GlcNAc labelling patterns in both WT N2 and *aex-3*p::Tau(V337M) strain [37], this proteomic analysis describes the sets of O-GlcNAcylated proteins detected in each strain. While previous studies have suggested functional coupling between tau phosphorylation and O-GlcNAcylation through OGT-dependent competition, with proposed consequences for splicing and proteostasis during expression of Alzheimer’s disease-associated tau variants [38,39,4,5], the present study was designed to identify O-GlcNAc-modified protein targets rather than to quantify regulatory interactions or modification dynamics. Accordingly, the data are interpreted in a descriptive framework, emphasizing protein identification and contextual annotation. The integration of quantitative O-GlcNAc stoichiometry, OGT/OGA activity measurements, and tau phosphorylation analyses will be necessary to assess the coordination among these processes in this model directly. The identified datasets provide a resource for future studies aimed at characterizing O-GlcNAcylated proteins and investigating their roles across development, adulthood, stress-associated conditions, and tau-related *C. elegans* models relevant to AD.

## Supporting information

S1 Table List of annotated O-glycopeptides identified from samples of WT N2 and of *aex-3* transgenic strain and corresponding human orthologs in UniProt Database. (CSV)

S2 Table Orthology-based gene mapping between O-glycopeptides from WT N2 and *aex-3* transgenic strain and human genes. (CSV)

S1 Dataset Extracted ion annotations obtained from the nano-LC-MS/MS analysis for each strain. (RAR)

S2 Dataset Peptide matching from Mascot Distiller search results. (PDF)

S3 Dataset Term enrichments data of the STRING-based PPI analysis of O-GlcNAcylated proteins identified in WT N2 (21 first-shell interactions) and in *aex-3*p::Tau(V337M) strain (1 first-shell interaction). (XLSX)

S4 Dataset Term enrichments data of the STRING-based PPI analysis of O-GlcNAcylated proteins identified in *aex-3*p::Tau(V337M) strain (21 first- and second-shell interactions). (XLSX)

S1 Fig Bubble charts of term enrichments of the STRING-based PPI analysis of O-GlcNAcylated proteins identified in the *aex-3*p::Tau(V337M) AD Model (21 first- and second-shell interactions). (TIF)
