# Supplementary material for: Proteome-based investigation of O-GlcNAcylation in a C. elegans model of ageing and Alzheimer’s disease: Functional support for earlier hypothesis-generating findings
Source: PLoS One. 2026 Mar 30;21(3):e0344865. doi: 10.1371/journal.pone.0344865 (PMC13035170; doi:10.1371/journal.pone.0344865)
Supplement: S2 Dataset — (PDF) [file pone.0344865.s004.pdf]

Mascot Search Results

Peptide View

MS/MS Fragmentation of **PNSRHDNVSPSK**
Found in **G5EET4** in **UP1940_C_elegans**, Glutamic acid-rich protein OS=Caenorhabditis elegans OX=6239 GN=CELE_F40G12.11 PE=1 SV=1

Match to Query 2295: 1742.813688 from(872.414120,2+) intensity(212588.84) scans(40241) rawscans(sn40241) rtinseconds(13366.562) index(1545)
Title: 1546: Scan 40241 (rt=13366.6) [D:\L1N2-01.raw]
Data file L1N2-01.temp.mgf

observedPNy11a1Sy10a2Ry9a3Hy8a4Dy7a5Ny6a6Vy5a7HESy4a8Py3a9HESy2a10Ky1a11y(11)-4061240.5968 | 4.9 ppmy(9)-4061039.5260 | 1.9 ppmy(10)-4061126.5592 | 0.7 ppma(8)892.4515 | -14.7 ppma(7)793.3872 | -21.8 ppma(5)564.3183 | -32.3 ppma(6)679.3577 | -45.1 ppmZoom...600800100012001400m/z020406080100% of base peak0500100015002000250030003500ion current

to

Monoisotopic mass of neutral peptide Mr(calc): 1742.8071
Fixed modifications: Carbamidomethyl (C) (apply to specified residues or termini only)
Variable modifications:
S9 : HexNAc (ST), with neutral losses 203.0794(shown in table), 0.0000
S11 : HexNAc (ST), with neutral losses 203.0794(shown in table), 0.0000
Ions Score: 41 **Expect:** 0.00093
Peak matches: 7/192 fragment ions using 8 most intense peaks
Annotated fragments: 7/192 (help)

#	a	a++	a*	a*++	b	b++	b*	b*++	Seq.	y	y++	y*	y*++	#
1	70.0651	35.5362			98.0600	49.5337			P					12
2	184.1081	92.5577	167.0815	84.0444	212.1030	106.5551	195.0764	98.0418	N	*1240.6029*	620.8051	1223.5763	612.2918	11
3	271.1401	136.0737	254.1135	127.5604	299.1350	150.0711	282.1084	141.5579	S	*1126.5600*	563.7836	1109.5334	555.2703	10
4	427.2412	214.1242	410.2146	205.6110	455.2361	228.1217	438.2096	219.6084	R	*1039.5279*	520.2676	1022.5014	511.7543	9
5	*564.3001*	282.6537	547.2736	274.1404	592.2950	296.6511	575.2685	288.1379	H	883.4268	442.2170	866.4003	433.7038	8
6	*679.3270*	340.1672	662.3005	331.6539	707.3220	354.1646	690.2954	345.6513	D	746.3679	373.6876	729.3414	365.1743	7
7	*793.3700*	397.1886	776.3434	388.6754	821.3649	411.1861	804.3383	402.6728	N	631.3410	316.1741	614.3144	307.6608	6
8	*892.4384*	446.7228	875.4118	438.2096	920.4333	460.7203	903.4068	452.2070	V	517.2980	259.1527	500.2715	250.6394	5
9	979.4704	490.2388	962.4439	481.7256	1007.4653	504.2363	990.4388	495.7230	S	418.2296	209.6184	401.2031	201.1052	4
10	1076.5232	538.7652	1059.4966	530.2520	1104.5181	552.7627	1087.4915	544.2494	P	331.1976	166.1024	314.1710	157.5892	3
11	1163.5552	582.2812	1146.5287	573.7680	1191.5501	596.2787	1174.5236	587.7654	S	234.1448	117.5761	217.1183	109.0628	2
12									K	147.1128	74.0600	130.0863	65.5468	1

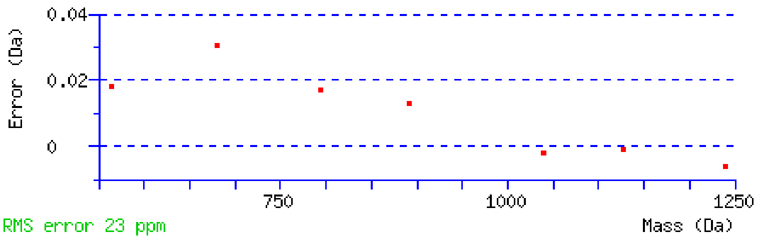

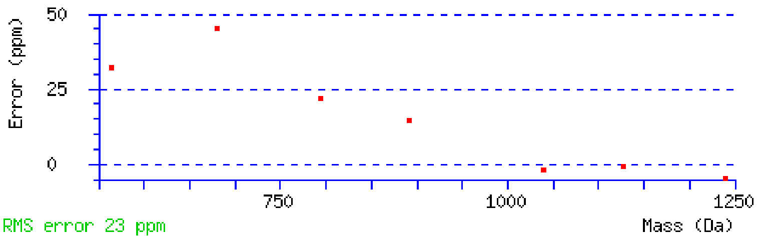

NCBI **BLAST** search of PNSRHDNVSPSK
(Parameters: blastp, nr protein database, expect=20000, no filter, PAM30)
Other BLAST web gateways

All matches to this query

Score	Mr(calc)	Delta	Sequence	Site Analysis

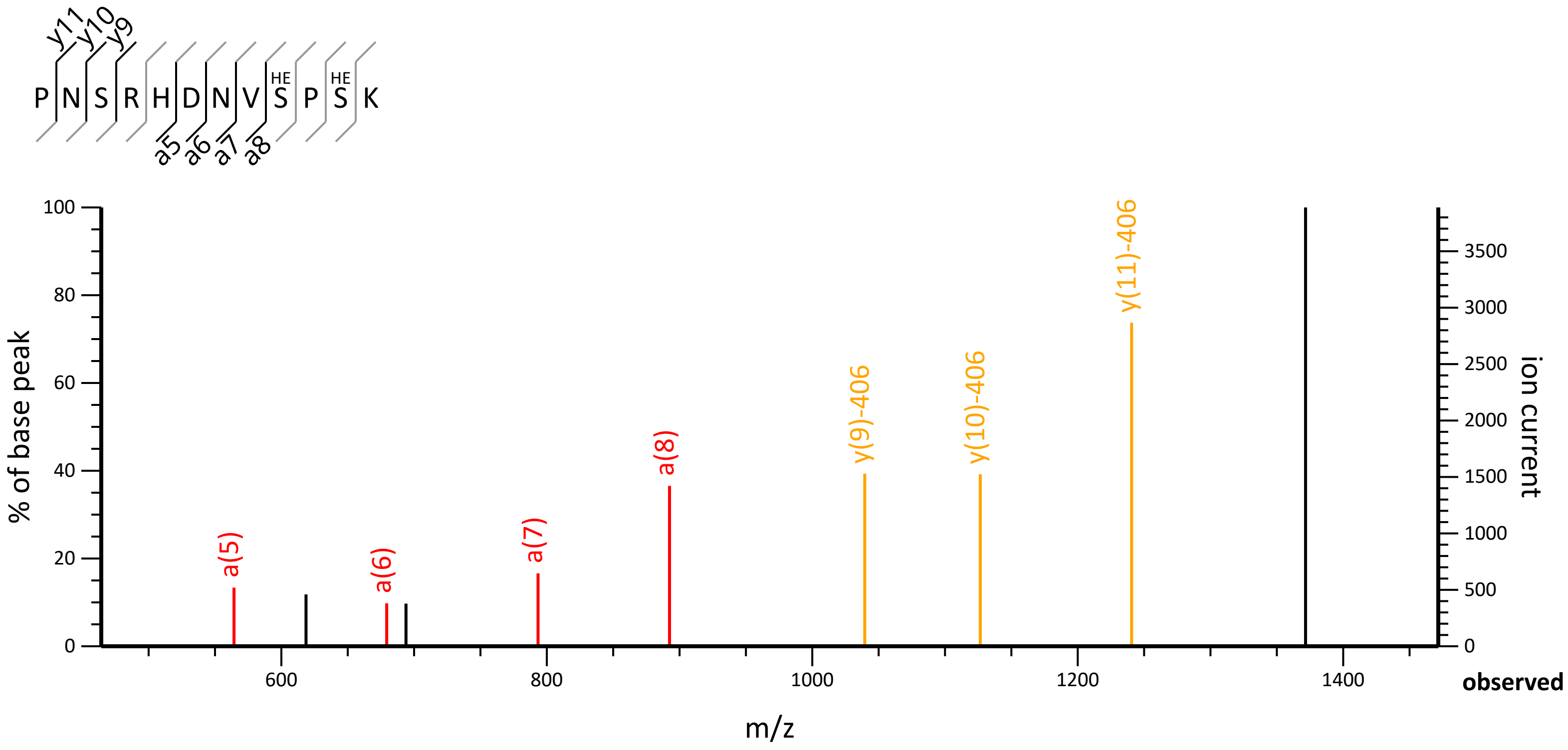

P N S R H D N V S P S K
HE
HE
y11
y10
y9
a5
a6
a7
a8
% of base peak
ion current
m/z
observed
a(5)
a(6)
a(7)
a(8)
y(9)-406
y(10)-406
y(11)-406
0
20
40
60
80
100
500
1000
1500
2000
2500
3000
3500
600
800
1000
1200
1400

40.6	1742.8071	0.0066	PNSRHDNVSPSK	HexNAc S9, S11 33.33%
40.6	1742.8071	0.0066	PNSRHDNVSPSK	HexNAc S3, S11 33.33%
40.6	1742.8071	0.0066	PNSRHDNVSPSK	HexNAc S3, S9 33.33%
7.7	1740.8539	1.9598	SKGIPICMVTSGGYQK	
6.9	1742.7974	0.0163	NWMSNVAWEFLK	
6.5	1740.7836	2.0301	KRTDSEMSQEPSK	
6.5	1740.7836	2.0301	KRTDSEMSQEPSK	
5.6	1740.8505	1.9632	QMQLTVFYHISR	
3.4	1741.8192	0.9945	KDQMSTEEQKDLYK	
3.3	1742.8397	-0.0260	VYLTSQLSEMTPR	

Mascot: http://www.matrixscience.com/

MATRIX SCIENCE Mascot Search Results

Peptide View

MS/MS Fragmentation of **RARDSASSSSSHSK**
Found in **CWC15_CAEEL** in **SwissProt**, Spliceosome-associated protein CWC15 homolog OS=Caenorhabditis elegans OX=6239 GN=cwc-15 PE=1 SV=1

Match to Query 4021: 2479.047912 from(827.356580,3+) intensity(86791.75) scans(14838) rawscans(sn14838) rtinseconds(5599.9938) index(26)
Title: 27: Scan 14838 (rt=5599.99) [D:\L1N2-01.raw]
Data file L1N2-01.temp.mgf

observedRAy13a1Ry12a2Dy11a3Sy10a4Ay9a5Sy8a6HESy7a7HESy6a8HESy5a9HESy4a10Hy3a11HESy2a12Ky1a13a(11)-8121064.4712 | 34.5 ppma(7)716.3278 | 72.7 ppma(10)-609977.4445 | 32.1 ppma(6)629.2895 | 92.6 ppmy(5)-609545.3331 | -119.8 ppmZoom...6008001000120014001600m/z020406080100% of base peak02004006008001000ion current

to

Monoisotopic mass of neutral peptide Mr(calc): 2477.0889
Fixed modifications: Carbamidomethyl (C) (apply to specified residues or termini only)
Variable modifications:
S8 : HexNAc (ST), with neutral losses 203.0794(shown in table), 0.0000
S9 : HexNAc (ST), with neutral losses 203.0794(shown in table), 0.0000
S10 : HexNAc (ST), with neutral losses 203.0794(shown in table), 0.0000
S11 : HexNAc (ST), with neutral losses 203.0794(shown in table), 0.0000
S13 : HexNAc (ST), with neutral losses 203.0794(shown in table), 0.0000
Ions Score: 23 **Expect:** 0.018
Peak matches: 4/252 fragment ions using 6 most intense peaks
Annotated fragments: 5/252 (help)

#	a	a++	a*	a*++	b	b++	b*	b*++	Seq.	y	y++	y*	y*++	#
1	129.1135	65.0604	112.0869	56.5471	157.1084	79.0578	140.0818	70.5446	R					14
2	200.1506	100.5789	183.1240	92.0657	228.1455	114.5764	211.1190	106.0631	A	1306.5982	653.8027	1289.5716	645.2895	13
3	356.2517	178.6295	339.2251	170.1162	384.2466	192.6269	367.2201	184.1137	R	1235.5611	618.2842	1218.5345	609.7709	12
4	471.2786	236.1430	454.2521	227.6297	499.2736	250.1404	482.2470	241.6271	D	1079.4600	540.2336	1062.4334	531.7203	11
5	558.3107	279.6590	541.2841	271.1457	586.3056	293.6564	569.2790	285.1432	S	964.4330	482.7202	947.4065	474.2069	10
6	***629.3478***	315.1775	612.3212	306.6643	657.3427	329.1750	640.3161	320.6617	A	877.4010	439.2041	860.3744	430.6909	9
7	***716.3798***	358.6935	699.3533	350.1803	744.3747	372.6910	727.3482	364.1777	S	806.3639	403.6856	789.3373	395.1723	8
8	803.4118	402.2096	786.3853	393.6963	831.4068	416.2070	814.3802	407.6937	S	719.3319	360.1696	702.3053	351.6563	7
9	890.4439	445.7256	873.4173	437.2123	918.4388	459.7230	901.4122	451.2098	S	632.2998	316.6536	615.2733	308.1403	6
10	***977.4759***	489.2416	960.4493	480.7283	1005.4708	503.2390	988.4443	494.7258	S	545.2678	273.1375	528.2413	264.6243	5
11	***1064.5079***	532.7576	1047.4814	524.2443	1092.5028	546.7551	1075.4763	538.2418	S	458.2358	229.6215	441.2092	221.1082	4
12	1201.5668	601.2871	1184.5403	592.7738	1229.5617	615.2845	1212.5352	606.7712	H	371.2037	186.1055	354.1772	177.5922	3
13	1288.5989	644.8031	1271.5723	636.2898	1316.5938	658.8005	1299.5672	650.2873	S	234.1448	117.5761	217.1183	109.0628	2
14									K	147.1128	74.0600	130.0863	65.5468	1

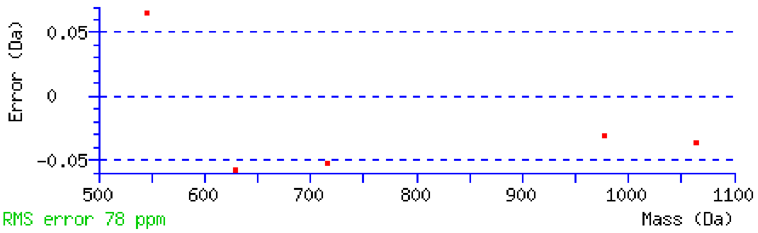

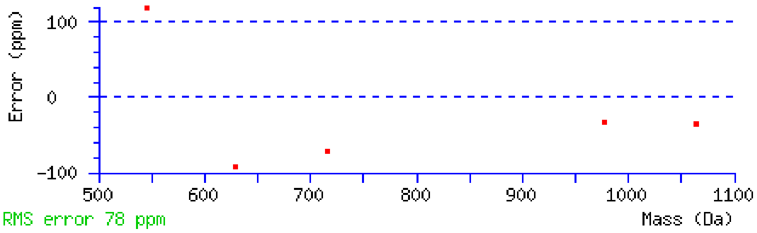

NCBI **BLAST** search of RARDSASSSSSHSK
(Parameters: blastp, nr protein database, expect=20000, no filter, PAM30)
Other BLAST web gateways

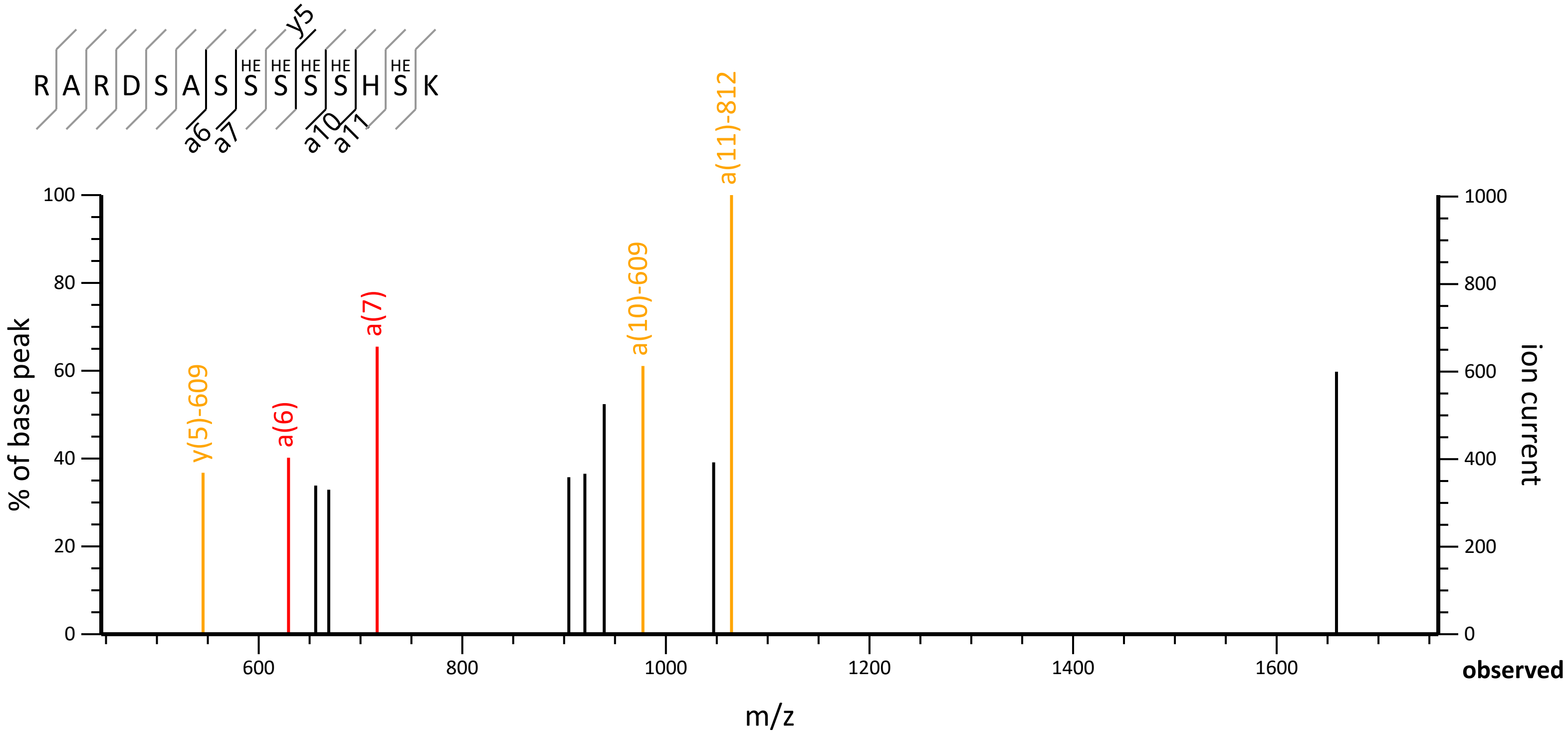

R A R D S A S S S S S H S K
HE HE HE HE HE
y5
a6 a7 a10 a11
y(5)-609
a(6)
a(7)
a(10)-609
a(11)-812
% of base peak
ion current
m/z
observed
0
20
40
60
80
100
200
400
600
800
1000
1200
1400
1600

All matches to this query

Score	Mr(calc)	Delta	Sequence	Site Analysis
22.9	2477.0889	1.9590	RARDSASSSSSHSK	HexNAc S8, S9, S10, S11, S13 5.34%
22.9	2477.0889	1.9590	RARDSASSSSSHSK	HexNAc S7, S9, S10, S11, S13 5.34%
22.9	2477.0889	1.9590	RARDSASSSSSHSK	HexNAc S7, S8, S10, S11, S13 5.34%
22.9	2477.0889	1.9590	RARDSASSSSSHSK	HexNAc S7, S8, S9, S11, S13 5.34%
22.9	2477.0889	1.9590	RARDSASSSSSHSK	HexNAc S7, S8, S9, S10, S13 5.34%
22.9	2477.0889	1.9590	RARDSASSSSSHSK	HexNAc S7, S8, S9, S10, S11 5.34%
22.9	2477.0889	1.9590	RARDSASSSSSHSK	HexNAc S5, S9, S10, S11, S13 5.34%
22.9	2477.0889	1.9590	RARDSASSSSSHSK	HexNAc S5, S8, S10, S11, S13 5.34%
22.9	2477.0889	1.9590	RARDSASSSSSHSK	HexNAc S5, S8, S9, S11, S13 5.34%
22.9	2477.0889	1.9590	RARDSASSSSSHSK	HexNAc S5, S8, S9, S10, S13 5.34%

Mascot: http://www.matrixscience.com/

Mascot Search Results

Peptide View

MS/MS Fragmentation of **TFDFRADKILESLTNSLK**
Found in **Q20127** in **UP1940_C_elegans**, NADP-dependent oxidoreductase domain-containing protein OS=Caenorhabditis elegans OX=6239 GN=mec-14 PE=4 SV=2

Match to Query 3668: 2301.224472 from(768.082100,3+) intensity(520592.22) scans(40707) rawscans(sn40707) rtinseconds(13456.88) index(1710)
Title: 1711: Scan 40707 (rt=13456.9) [D:\L1N2-01.raw]
Data file L1N2-01.temp.mgf

observedTFy17L1Dy16L2Fy15L3Ry14L4Ay13L5Dy12L6Ky11L7Iy10L8Ly9L9Ey8L10Sy7L11Ly6L12Ty5L13Ny4L14HES y3L15Ly2L16Ky1L17y(7)-203762.4382 | -3.4 ppmy*(6)861.5020 | -52.9 ppmy(10)-2031117.6141 | 28.8 ppmy(5)-203562.3251 | -10.0 ppmy(6)-203675.4118 | -12.1 ppmZoom...500600700800900100011001200m/z020406080100% of base peak0500100015002000250030003500ion current

to

Monoisotopic mass of neutral peptide Mr(calc): 2300.1900
Fixed modifications: Carbamidomethyl (C) (apply to specified residues or termini only)
Variable modifications:
S16 : HexNAc (ST), with neutral losses 203.0794(shown in table), 0.0000
Ions Score: 22 **Expect:** 0.046
Peak matches: 4/264 fragment ions using 6 most intense peaks
Annotated fragments: 5/264 (help)

#	a	a++	a*	a*++	b	b++	b*	b*++	Seq.	y	y++	y*	y*++	#
1	74.0600	37.5337			102.0550	51.5311			T					18
2	221.1285	111.0679			249.1234	125.0653			F	1997.0702	999.0387	1980.0437	990.5255	17
3	336.1554	168.5813			364.1503	182.5788			D	1850.0018	925.5045	1832.9753	916.9913	16
4	483.2238	242.1155			511.2187	256.1130			F	1734.9749	867.9911	1717.9483	859.4778	15
5	639.3249	320.1661	622.2984	311.6528	667.3198	334.1636	650.2933	325.6503	R	1587.9064	794.4569	1570.8799	785.9436	14
6	710.3620	355.6847	693.3355	347.1714	738.3570	369.6821	721.3304	361.1688	A	1431.8053	716.4063	1414.7788	707.8930	13
7	825.3890	413.1981	808.3624	404.6849	853.3839	427.1956	836.3573	418.6823	D	1360.7682	680.8877	1343.7417	672.3745	12
8	953.4839	477.2456	936.4574	468.7323	981.4789	491.2431	964.4523	482.7298	K	1245.7413	623.3743	1228.7147	614.8610	11
9	1066.5680	533.7876	1049.5415	525.2744	1094.5629	547.7851	1077.5364	539.2718	I	***1117.6463***	559.3268	1100.6198	550.8135	10
10	1179.6521	590.3297	1162.6255	581.8164	1207.6470	604.3271	1190.6204	595.8139	L	1004.5623	502.7848	987.5357	494.2715	9
11	1308.6947	654.8510	1291.6681	646.3377	1336.6896	668.8484	1319.6630	660.3352	E	891.4782	446.2427	874.4516	437.7295	8
12	1395.7267	698.3670	1378.7001	689.8537	1423.7216	712.3644	1406.6951	703.8512	S	***762.4356***	381.7214	745.4090	373.2082	7
13	1508.8108	754.9090	1491.7842	746.3957	1536.8057	768.9065	1519.7791	760.3932	L	***675.4036***	338.2054	658.3770	329.6921	6
14	1609.8584	805.4329	1592.8319	796.9196	1637.8533	819.4303	1620.8268	810.9170	T	***562.3195***	281.6634	545.2930	273.1501	5
15	1723.9014	862.4543	1706.8748	853.9410	1751.8963	876.4518	1734.8697	867.9385	N	461.2718	231.1395	444.2453	222.6263	4
16	1810.9334	905.9703	1793.9068	897.4571	1838.9283	919.9678	1821.9018	911.4545	S	347.2289	174.1181	330.2023	165.6048	3
17	1924.0175	962.5124	1906.9909	953.9991	1952.0124	976.5098	1934.9858	967.9965	L	260.1969	130.6021	243.1703	122.0888	2
18									K	147.1128	74.0600	130.0863	65.5468	1

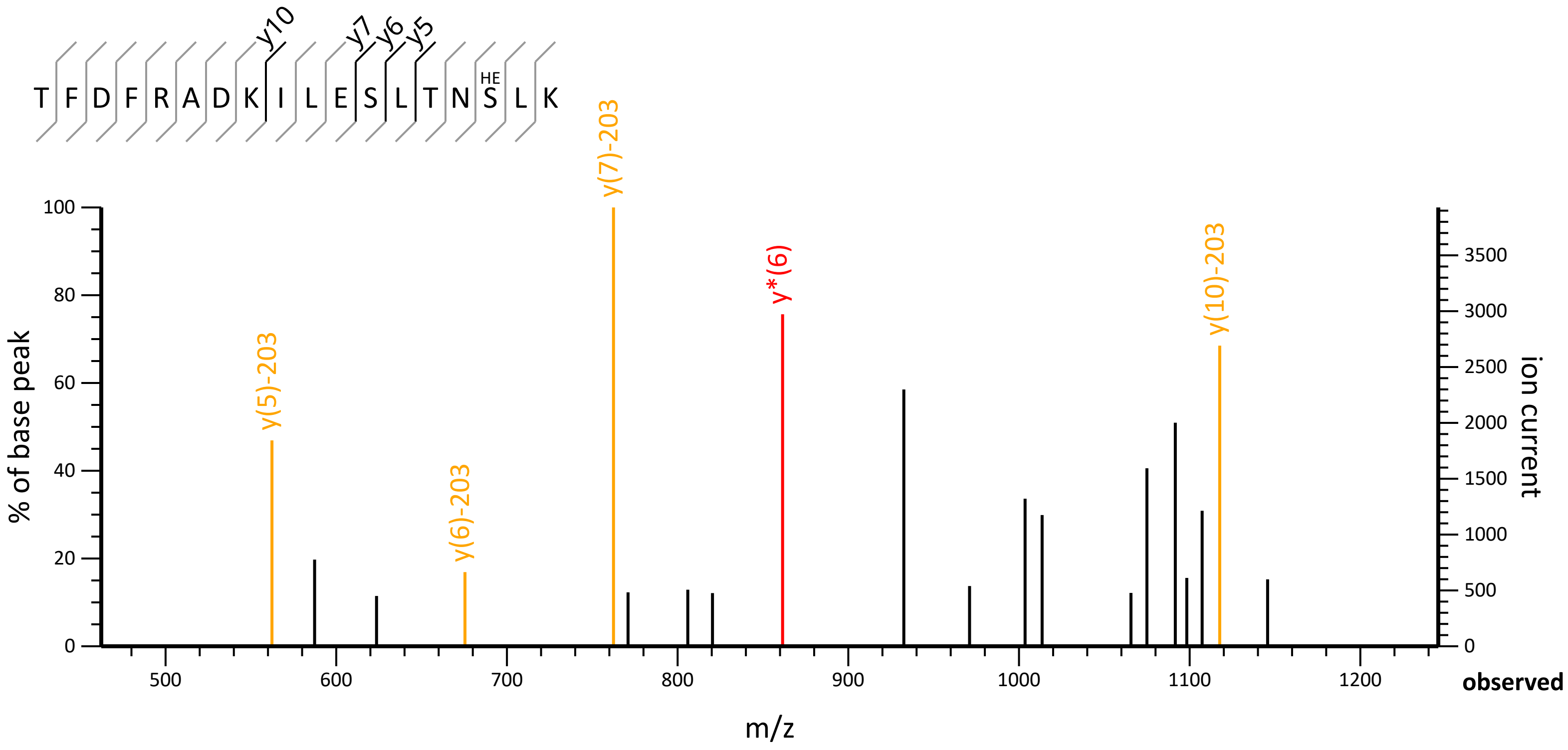

T F D F R A D K I L E S L T N S L K
HE
y10
y7
y6
y5
y(5)-203
y(6)-203
y(7)-203
y*(6)
y(10)-203
% of base peak
ion current
m/z
observed

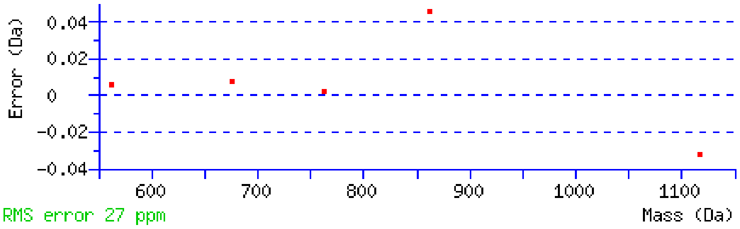

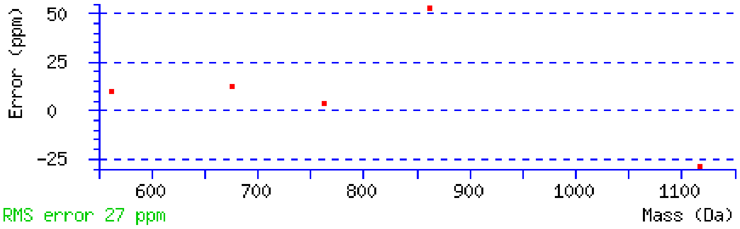

NCBI **BLAST** search of TFDFRADKILESLTNSLK
(Parameters: blastp, nr protein database, expect=20000, no filter, PAM30)
Other BLAST web gateways

All matches to this query

Score	Mr(calc)	Delta	Sequence	Site Analysis
22.2	2300.1900	1.0345	TFDFRADKILESLTNSLK	HexNAc S16 25.00%
22.2	2300.1900	1.0345	TFDFRADKILESLTNSLK	HexNAc T14 25.00%
22.2	2300.1900	1.0345	TFDFRADKILESLTNSLK	HexNAc S12 25.00%
22.2	2300.1900	1.0345	TFDFRADKILESLTNSLK	HexNAc T1 25.00%
7.5	2299.2172	2.0073	SPILSTIAESIHGASSIRAFDK	
7.2	2300.2263	0.9982	LEQLILKNSEELESLQTWK	
7.2	2301.2117	0.0128	NAVYNLSLSTLSVNHKIDWK	
5.4	2301.1939	0.0305	KRSMSLWVEFITASGYLSAR	
4.7	2301.1965	0.0280	ADSILFKTRLPQNHQK	
4.6	2299.2199	2.0046	DLFITSLIDTIVKPYR	

Mascot: http://www.matrixscience.com/

MATRIX SCIENCE Mascot Search Results

Peptide View

MS/MS Fragmentation of **VGLIAARRTGR**
Found in **RL8_CAEEL** in **SwissProt**, Large ribosomal subunit protein uL2 OS=Caenorhabditis elegans OX=6239 GN=rpl-8 PE=1 SV=1

Match to Query 1744: 1373.829788 from(687.922170,2+) intensity(267442.41) scans(42165) rawscans(sn42165) rtinseconds(13713.183) index(2231)
Title: 2232: Scan 42165 (rt=13713.2) [D:\L1N2-01.raw]
Data file L1N2-01.temp.mgf

observedVGy10L1Ly9L2Iy8L3Ay7L4Ay6L5Ry5L6Ry4L7HETy3L8Gy2L9Ry1L10y(7)990.5956 | -52.1 ppmy(6)919.5570 | -54.5 ppmy(5)848.5248 | -65.0 ppmy(8)1103.6871 | -53.6 ppmZoom...6007008009001000110012000m/z020406080100% of base peak050010001500ion current

to

Monoisotopic mass of neutral peptide Mr(calc): 1371.7946
Fixed modifications: Carbamidomethyl (C) (apply to specified residues or termini only)
Variable modifications:
T9 : HexNAc (ST), with neutral losses 0.0000(shown in table), 203.0794
Ions Score: 22 **Expect:** 0.018
Peak matches: 4/144 fragment ions using 8 most intense peaks
Annotated fragments: 4/144 (help)

#	a	a++	a*	a*++	b	b++	b*	b*++	Seq.	y	y++	y*	y*++	#
1	72.0808	36.5440			100.0757	50.5415			V					11
2	129.1022	65.0548			157.0972	79.0522			G	1273.7335	637.3704	1256.7070	628.8571	10
3	242.1863	121.5968			270.1812	135.5942			L	1216.7120	608.8597	1199.6855	600.3464	9
4	355.2704	178.1388			383.2653	192.1363			I	***1103.6280***	552.3176	1086.6014	543.8044	8
5	426.3075	213.6574			454.3024	227.6548			A	***990.5439***	495.7756	973.5174	487.2623	7
6	497.3446	249.1759			525.3395	263.1734			A	***919.5068***	460.2570	902.4803	451.7438	6
7	653.4457	327.2265	636.4192	318.7132	681.4406	341.2239	664.4141	332.7107	R	***848.4697***	424.7385	831.4431	416.2252	5
8	809.5468	405.2770	792.5203	396.7638	837.5417	419.2745	820.5152	410.7612	R	692.3686	346.6879	675.3420	338.1747	4
9	1113.6739	557.3406	1096.6473	548.8273	1141.6688	571.3380	1124.6422	562.8248	T	536.2675	268.6374	519.2409	260.1241	3
10	1170.6953	585.8513	1153.6688	577.3380	1198.6902	599.8488	1181.6637	591.3355	G	232.1404	116.5738	215.1139	108.0606	2
11									R	175.1190	88.0631	158.0924	79.5498	1

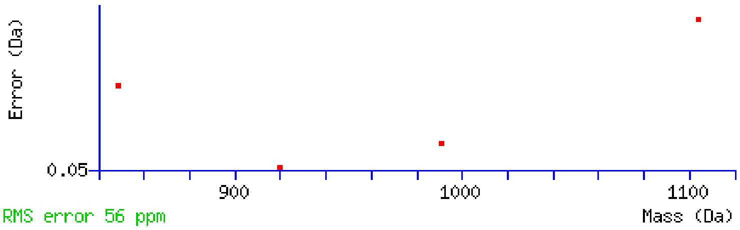

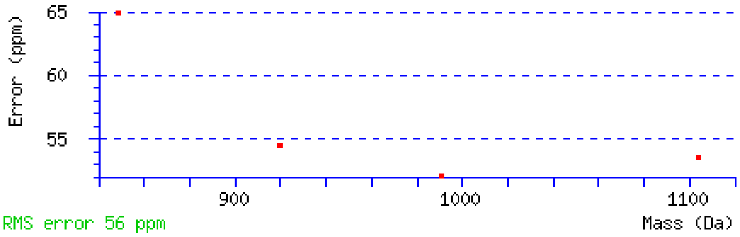

NCBI **BLAST** search of VGLIAARRTGR
(Parameters: blastp, nr protein database, expect=20000, no filter, PAM30)
Other BLAST web gateways

All matches to this query

Score	Mr(calc)	Delta	Sequence
22.1	1371.7946	2.0352	VGLIAARRTGR
3.4	1372.8038	1.0260	AKVRQLVTQK
3.0	1373.7918	0.0379	LKTLFLAHTK

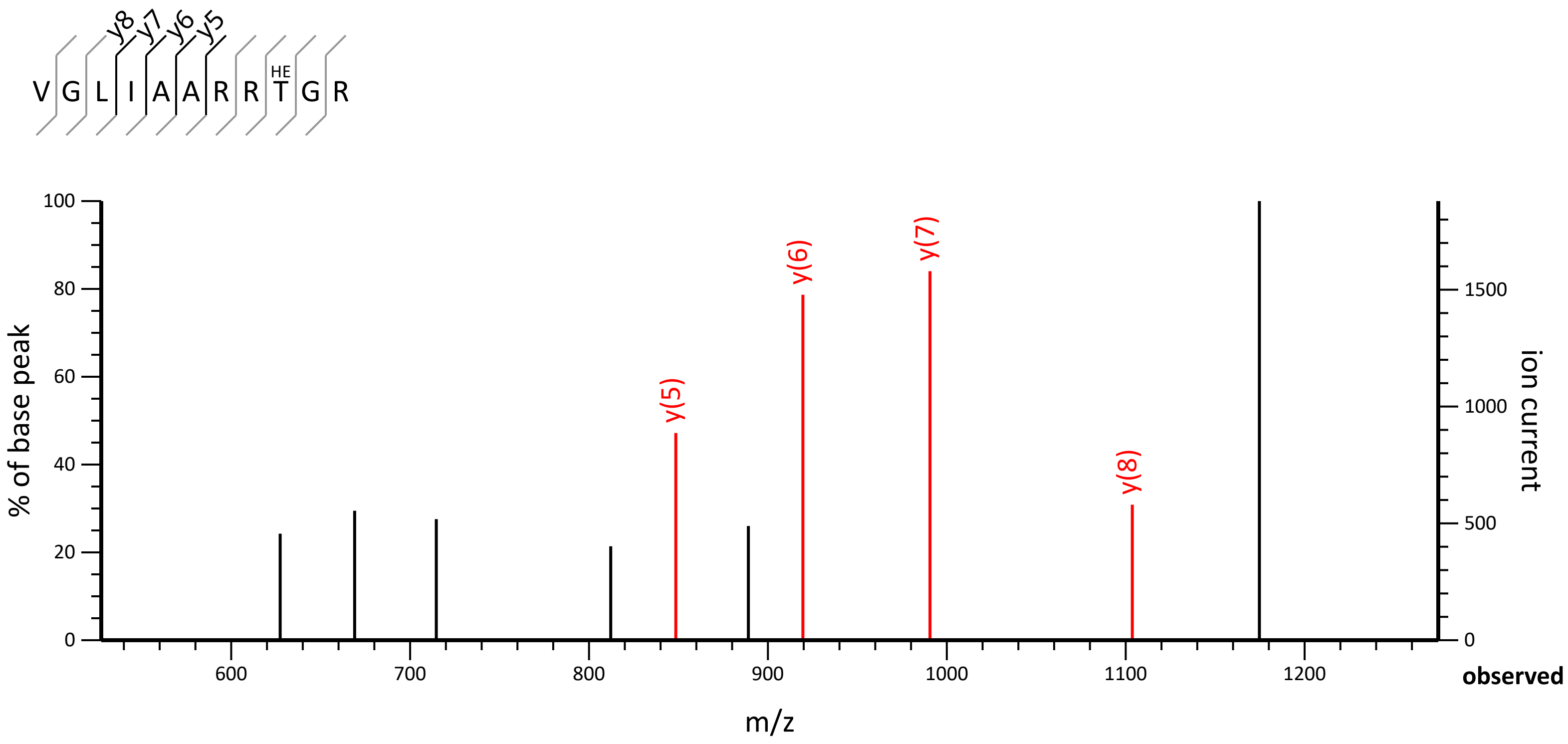

y8
y7
y6
y5
V G L I A A R R T G R
HE
y(5)
y(6)
y(7)
y(8)
% of base peak
ion current
m/z
observed
100
80
60
40
20
0
1500
1000
500
600
700
800
900
1000
1100
1200

2.0	1371.8020	2.0278	VVAVKTCRIAQK
1.5	1373.8395	-0.0097	IGTIVNLLPAVHK
0.7	1371.8020	2.0278	RVLMLPSVASKR

Mascot: http://www.matrixscience.com/

MATRIX SCIENCE Mascot Search Results

Peptide View

MS/MS Fragmentation of **MFITRGLILISLLFVFVMTDDTHDK**
Found in **Q4R114** in **UP1940_C_elegans**, PIR protein OS=Caenorhabditis elegans OX=6239 GN=CELE_F56D6.12 PE=4 SV=1

Match to Query 5352: 3346.667772 from(1116.563200,3+) intensity(372682.34) scans(41848) rawscans(sn41848) rtinseconds(13659.149) index(2116)
Title: 2117: Scan 41848 (rt=13659.1) [D:\L1N2-01.raw]
Data file L1N2-01.temp.mgf

observedMFy24b1Iy23b2Ty22b3Ry21b4Gy20b5Ly19b6Iy18b7Ly17b8Iy16b9Sy15b10Ly14b11Ly13b12Fy12b13Vy11b14F y10b15Vy9b16OXMy8b17HETy7b18Dy6b19Dy5b20HETy4b21Hy3b22Dy2b23Ky1b24y(15)$^{++}$1095.5348 | -21.2 ppmy(12)$^{++}$938.9871 | -80.5 ppmy(17)$^{++}$1208.6219 | -21.7 ppmb(13)$^{++}$736.3883 | 93.4 ppmy(11)$^{++}$865.4495 | -83.4 ppmZoom...6008001000120014001600m/z020406080100% of base peak010002000300040005000ion current

to

Monoisotopic mass of neutral peptide Mr(calc): 3346.7081
Fixed modifications: Carbamidomethyl (C) (apply to specified residues or termini only)
Variable modifications:
M18 : Oxidation (M), with neutral losses 0.0000(shown in table), 63.9983
T19 : HexNAc (ST), with neutral losses 0.0000(shown in table), 203.0794
T22 : HexNAc (ST), with neutral losses 0.0000(shown in table), 203.0794
Ions Score: 22 **Expect:** 0.026
Peak matches: 5/644 fragment ions using 6 most intense peaks
Annotated fragments: 5/644 (help)

#	a	a++	a*	a*++	b	b++	b*	b*++	Seq.	y	y++	y*	y*++	#
1	104.0528	52.5301			132.0478	66.5275			M					25
2	251.1213	126.0643			279.1162	140.0617			F	3216.6748	1608.8411	3199.6483	1600.3278	24
3	364.2053	182.6063			392.2002	196.6038			I	3069.6064	1535.3069	3052.5799	1526.7936	23
4	465.2530	233.1301			493.2479	247.1276			T	2956.5224	1478.7648	2939.4958	1470.2515	22
5	621.3541	311.1807	604.3276	302.6674	649.3490	325.1782	632.3225	316.6649	R	2855.4747	1428.2410	2838.4481	1419.7277	21
6	678.3756	339.6914	661.3490	331.1782	706.3705	353.6889	689.3439	345.1756	G	2699.3736	1350.1904	2682.3470	1341.6772	20
7	791.4596	396.2335	774.4331	387.7202	819.4546	410.2309	802.4280	401.7176	L	2642.3521	1321.6797	2625.3256	1313.1664	19
8	904.5437	452.7755	887.5172	444.2622	932.5386	466.7729	915.5121	458.2597	I	2529.2681	1265.1377	2512.2415	1256.6244	18
9	1017.6278	509.3175	1000.6012	500.8042	1045.6227	523.3150	1028.5961	514.8017	L	2416.1840	**1208.5956**	2399.1574	1200.0824	17
10	1130.7118	565.8596	1113.6853	557.3463	1158.7067	579.8570	1141.6802	571.3437	I	2303.0999	1152.0536	2286.0734	1143.5403	16
11	1217.7439	609.3756	1200.7173	600.8623	1245.7388	623.3730	1228.7122	614.8598	S	2190.0159	**1095.5116**	2172.9893	1086.9983	15
12	1330.8279	665.9176	1313.8014	657.4043	1358.8228	679.9151	1341.7963	671.4018	L	2102.9838	1051.9956	2085.9573	1043.4823	14
13	1443.9120	722.4596	1426.8854	713.9464	1471.9069	**736.4571**	1454.8804	727.9438	L	1989.8998	995.4535	1972.8732	986.9402	13
14	1590.9804	795.9938	1573.9539	787.4806	1618.9753	809.9913	1601.9488	801.4780	F	1876.8157	**938.9115**	1859.7892	930.3982	12
15	1690.0488	845.5280	1673.0223	837.0148	1718.0437	859.5255	1701.0172	851.0122	V	1729.7473	**865.3773**	1712.7207	856.8640	11
16	1837.1172	919.0623	1820.0907	910.5490	1865.1121	933.0597	1848.0856	924.5464	F	1630.6789	815.8431	1613.6523	807.3298	10
17	1936.1856	968.5965	1919.1591	960.0832	1964.1806	982.5939	1947.1540	974.0806	V	1483.6105	742.3089	1466.5839	733.7956	9
18	2083.2210	1042.1142	2066.1945	1033.6009	2111.2160	1056.1116	2094.1894	1047.5983	M	1384.5420	692.7747	1367.5155	684.2614	8
19	2387.3481	1194.1777	2370.3215	1185.6644	2415.3430	1208.1751	2398.3165	1199.6619	T	1237.5066	619.2570	1220.4801	610.7437	7
20	2502.3750	1251.6912	2485.3485	1243.1779	2530.3700	1265.6886	2513.3434	1257.1753	D	933.3796	467.1934	916.3530	458.6802	6
21	2617.4020	1309.2046	2600.3754	1300.6914	2645.3969	1323.2021	2628.3703	1314.6888	D	818.3527	409.6800	801.3261	401.1667	5
22	2921.5290	1461.2682	2904.5025	1452.7549	2949.5240	1475.2656	2932.4974	1466.7523	T	703.3257	352.1665	686.2992	343.6532	4
23	3058.5879	1529.7976	3041.5614	1521.2843	3086.5829	1543.7951	3069.5563	1535.2818	H	399.1987	200.1030	382.1721	191.5897	3
24	3173.6149	1587.3111	3156.5883	1578.7978	3201.6098	1601.3085	3184.5833	1592.7953	D	262.1397	131.5735	245.1132	123.0602	2
25									K	147.1128	74.0600	130.0863	65.5468	1

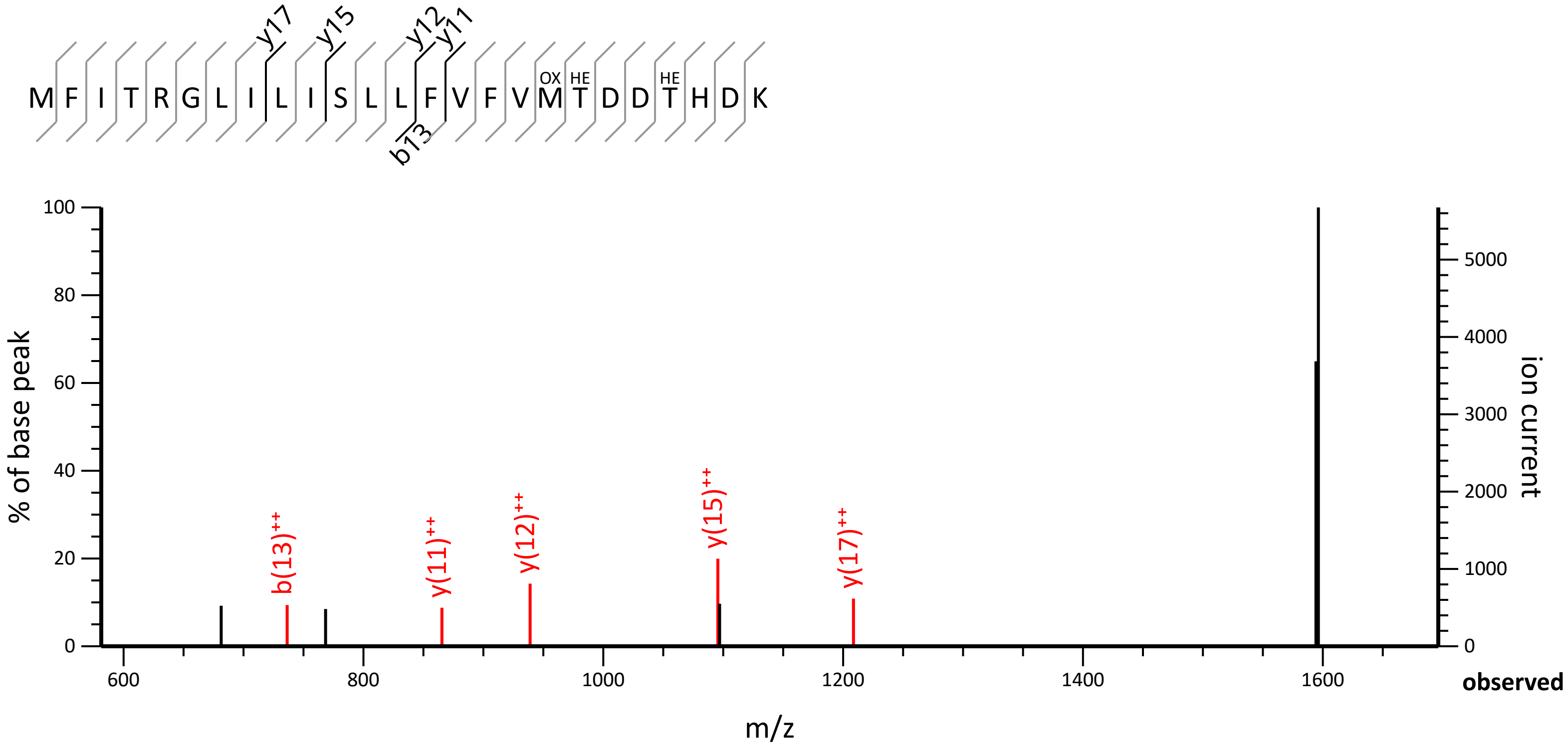

M F I T R G L I L I S L L F V F V M T D D T H D K
OX HE HE
y17 y15 y12 y11 b13
% of base peak
ion current
m/z
observed
b(13)++
y(11)++
y(12)++
y(15)++
y(17)++

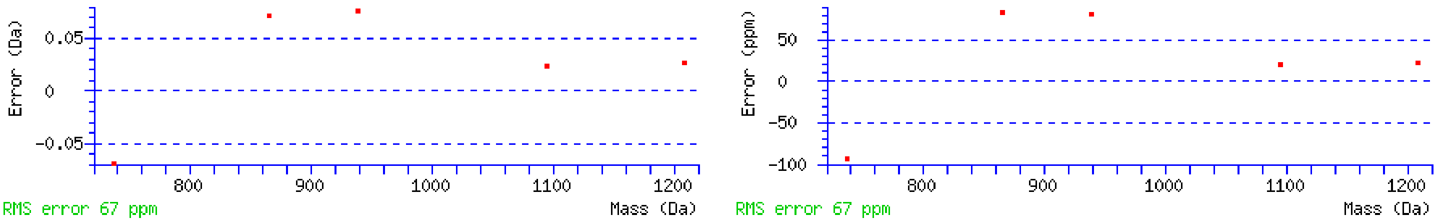

NCBI **BLAST** search of [MFITRGLILISLLFVFVMTDDTHDK]
(Parameters: blastp, nr protein database, expect=20000, no filter, PAM30)
Other BLAST [web gateways]

All matches to this query

Score	Mr(calc)	Delta	Sequence	Site Analysis
22.1	3346.7081	-0.0403	MFITRGLILISLLFVFVMTDDTHDK	HexNAc T19, T22, Oxidation M18; 91.90%
5.0	3346.6780	-0.0102	RTSHLIKDILDLPTVNGEIDEFGR	
3.2	3344.6333	2.0344	NSLISRPPTEKSMAPLGYIDDLYR	
3.2	3344.6333	2.0344	NSLISRPPTEKSMAPLGYIDDLYR	
3.2	3344.6333	2.0344	NSLISRPPTEKSMAPLGYIDDLYR	
3.2	3344.6333	2.0344	NSLISRPPTEKSMAPLGYIDDLYR	
2.7	3344.6235	2.0443	HKYTNNENILVDHVEKVDPEVFDIMK	
2.7	3346.6941	-0.0264	RETLFLTMTVSNGFKAEELMWQSSVSLVK	
2.2	3344.6445	2.0232	SADIYSLAVIASEVLTRKEAWNMAER	
2.2	3344.6445	2.0232	SADIYSLAVIASEVLTRKEAWNMAER	

Mascot Search Results

Peptide View

MS/MS Fragmentation of **LLVADILACNDDTPASAMMAGNGPVATMSLQVK**
Found in **Q9BLA0** in **UP1940_C_elegans**, K Homology domain-containing protein OS=Caenorhabditis elegans OX=6239 GN=fubl-3 PE=1 SV=1

Match to Query 3660: 3520.028656 from(881.014440,4+) intensity(517471.72) scans(38392) rawscans(sn38392) rtinseconds(13966.695) index(1104)
Title: 1105: Scan 38392 (rt=13966.7) [D:\Adult_N2-02.raw]
Data file Adult_N2-02.temp.mgf

Click mouse within plot area to zoom in by factor of two about that point
Or, Plot from 550 to 1300 Da Full range
Show Y-axis

MS/MS spectrum of LLVADILACNDDTPASAMMAGNGPVATMSLQVK

Monoisotopic mass of neutral peptide Mr(calc): 3519.6969
Variable modifications:
S29 : HexNAc (ST), with neutral losses 0.0000(shown in table), 203.0794
Ions Score: 43 **Expect:** 0.011
Peak matches: 24/492 fragment ions using 19 most intense peaks
Annotated fragments: 26/492 (help)

#	a	a++	a*	a*++	b	b++	b*	b*++	Seq.	y	y++	y*	y*++	#
1	86.0964	43.5519			114.0913	57.5493			L					33
2	199.1805	100.0939			227.1754	114.0913			L	3407.6201	1704.3137	3390.5936	1695.8004	32
3	298.2489	149.6281			326.2438	163.6255			V	3294.5361	1647.7717	3277.5095	1639.2584	31
4	369.2860	185.1466			397.2809	199.1441			A	3195.4677	1598.2375	3178.4411	1589.7242	30
5	484.3130	242.6601			512.3079	256.6576			D	3124.4305	1562.7189	3107.4040	1554.2056	29
6	597.3970	299.2022			625.3919	313.1996			I	3009.4036	1505.2054	2992.3770	1496.6922	28
7	710.4811	355.7442			738.4760	369.7416			L	2896.3195	1448.6634	2879.2930	1440.1501	27
8	781.5182	391.2627			809.5131	405.2602			A	2783.2355	1392.1214	2766.2089	1383.6081	26
9	**884.5274**	442.7673			912.5223	456.7648			C	2712.1984	1356.6028	2695.1718	1348.0895	25
10	**998.5703**	499.7888	981.5438	491.2755	1026.5652	513.7863	1009.5387	505.2730	N	2609.1892	1305.0982	2592.1626	1296.5849	24
11	1113.5973	557.3023	1096.5707	548.7890	1141.5922	571.2997	1124.5656	562.7864	D	2495.1462	1248.0768	2478.1197	1239.5635	23
12	1228.6242	614.8157	1211.5977	606.3025	1256.6191	628.8132	1239.5926	620.2999	D	2380.1193	1190.5633	2363.0927	1182.0500	22
13	1329.6719	***665.3396***	1312.6453	656.8263	1357.6668	679.3370	1340.6402	670.8238	T	2265.0924	1133.0498	2248.0658	1124.5365	21
14	1426.7246	***713.8660***	1409.6981	705.3527	1454.7196	727.8634	1437.6930	719.3501	P	2164.0447	1082.5260	2147.0181	1074.0127	20
15	1497.7618	749.3845	1480.7352	740.8712	1525.7567	***763.3820***	1508.7301	754.8687	A	2066.9919	1033.9996	2049.9654	1025.4863	19
16	1584.7938	792.9005	1567.7672	784.3873	1612.7887	806.8980	1595.7622	798.3847	S	1995.9548	**998.4810**	1978.9282	989.9678	18
17	1655.8309	***828.4191***	1638.8044	819.9058	1683.8258	842.4165	1666.7993	833.9033	A	1908.9228	954.9650	1891.8962	946.4517	17

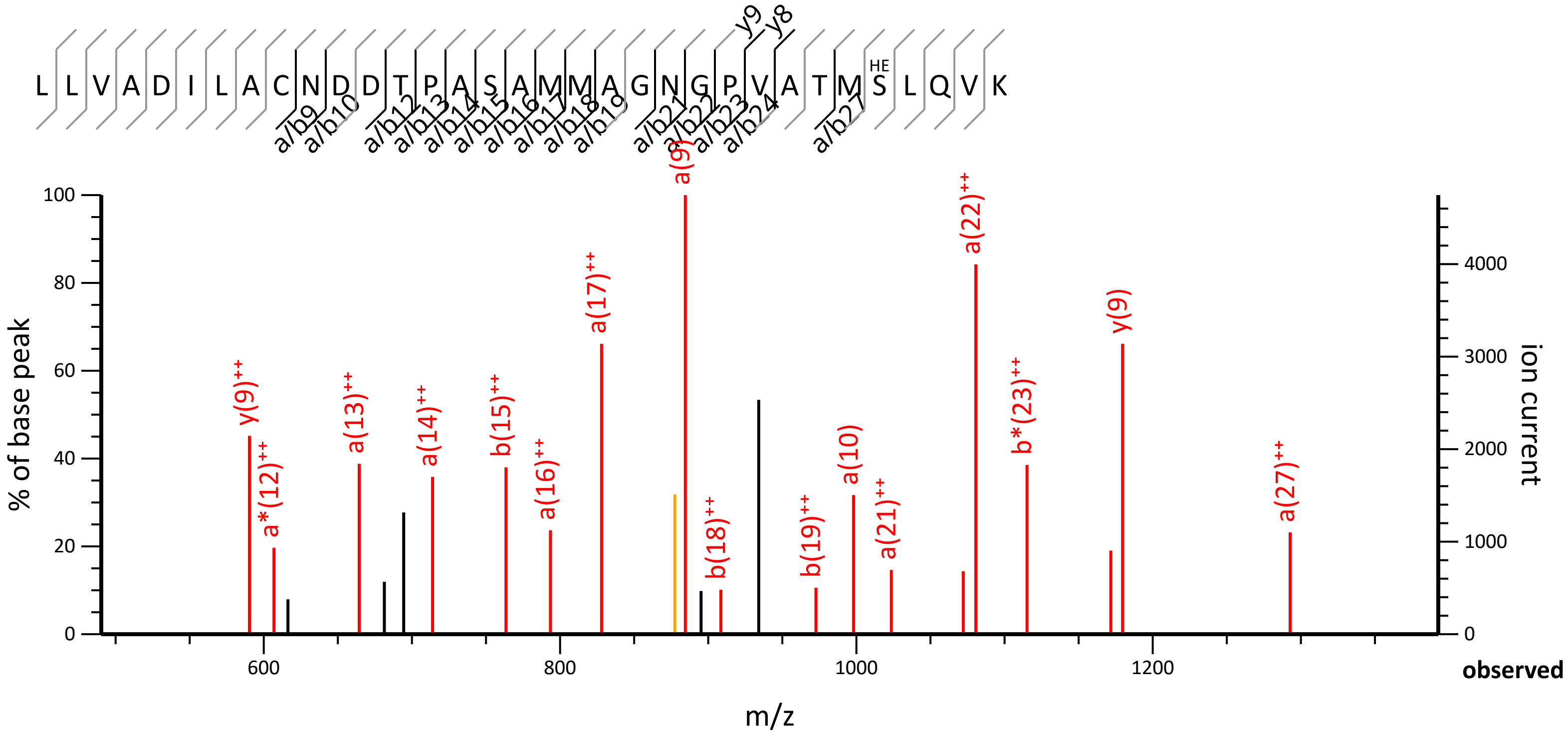

L L V A D I L A C N D D T P A S A M M A G N G P V A T M S L Q V K
HE
y9
y8
a/b9
a/b10
a/b12
a/b13
a/b14
a/b15
a/b16
a/b17
a/b18
a/b19
a/b21
a/b22
a/b23
a/b24
a/b27
y(9)++
a*(12)++
a(13)++
a(14)++
b(15)++
a(16)++
a(17)++
a(9)
b(18)++
b(19)++
a(10)
a(21)++
a(22)++
b*(23)++
y(9)
a(27)++
% of base peak
100
80
60
40
20
0
ion current
4000
3000
2000
1000
0
600
800
1000
1200
m/z
observed

**18**	1786.8714	893.9393	1769.8448	885.4261	1814.8663	***907.9368***	1797.8397	899.4235	**M**	1837.8857	919.4465	1820.8591	910.9332	**16**
**19**	1917.9119	959.4596	1900.8853	950.9463	1945.9068	***973.4570***	1928.8802	964.9438	**M**	1706.8452	853.9262	1689.8186	845.4129	**15**
**20**	1988.9490	994.9781	1971.9224	986.4649	2016.9439	1008.9756	1999.9173	1000.4623	**A**	1575.8047	788.4060	1558.7781	779.8927	**14**
**21**	2045.9704	***1023.4889***	2028.9439	1014.9756	2073.9654	1037.4863	2056.9388	1028.9730	**G**	1504.7676	752.8874	1487.7410	744.3741	**13**
**22**	2160.0134	***1080.5103***	2142.9868	1071.9971	2188.0083	1094.5078	2170.9817	1085.9945	**N**	1447.7461	724.3767	1430.7196	715.8634	**12**
**23**	2217.0348	1109.0211	2200.0083	1100.5078	2245.0298	1123.0185	2228.0032	1114.5052	**G**	1333.7032	667.3552	1316.6766	658.8420	**11**
**24**	2314.0876	1157.5474	2297.0611	1149.0342	2342.0825	***1171.5449***	2325.0560	1163.0316	**P**	1276.6817	638.8445	1259.6552	630.3312	**10**
**25**	2413.1560	1207.0816	2396.1295	1198.5684	2441.1509	1221.0791	2424.1244	1212.5658	**V**	**1179.6290**	**590.3181**	1162.6024	581.8048	**9**
**26**	2484.1931	1242.6002	2467.1666	1234.0869	2512.1880	1256.5977	2495.1615	1248.0844	**A**	**1080.5605**	540.7839	1063.5340	532.2706	**8**
**27**	2585.2408	***1293.1240***	2568.2143	1284.6108	2613.2357	1307.1215	2596.2092	1298.6082	**T**	1009.5234	505.2654	992.4969	496.7521	**7**
**28**	2716.2813	1358.6443	2699.2547	1350.1310	2744.2762	1372.6417	2727.2497	1364.1285	**M**	**908.4757**	454.7415	891.4492	446.2282	**6**
**29**	3006.3927	1503.7000	2989.3661	1495.1867	3034.3876	1517.6974	3017.3611	1509.1842	**S**	777.4353	389.2213	760.4087	380.7080	**5**
**30**	3119.4768	1560.2420	3102.4502	1551.7287	3147.4717	1574.2395	3130.4451	1565.7262	**L**	487.3239	244.1656	470.2973	235.6523	**4**
**31**	3247.5353	1624.2713	3230.5088	1615.7580	3275.5303	1638.2688	3258.5037	1629.7555	**Q**	374.2398	187.6235	357.2132	179.1103	**3**
**32**	3346.6038	1673.8055	3329.5772	1665.2922	3374.5987	1687.8030	3357.5721	1679.2897	**V**	246.1812	123.5942	229.1547	115.0810	**2**
**33**									**K**	147.1128	74.0600	130.0863	65.5468	**1**

Error Distribution

Error Distribution (ppm)

NCBI **BLAST** search of LLVADILACNDDTPASAMMAGNGPVATMSLQVK
(Parameters: blastp, nr protein database, expect=20000, no filter, PAM30)
Other BLAST web gateways

All matches to this query

Score	Mr(calc)	Delta	Sequence	Site Analysis
43.2	3519.6969	0.3318	LLVADILACNDDTPASAMMAGNGPVATMSLQVK	HexNAc S29 58.24%
37.2	3519.6969	0.3318	LLVADILACNDDTPASAMMAGNGPVATMSLQVK	HexNAc T27 14.33%
37.0	3519.6969	0.3318	LLVADILACNDDTPASAMMAGNGPVATMSLQVK	HexNAc S16 13.72%
37.0	3519.6969	0.3318	LLVADILACNDDTPASAMMAGNGPVATMSLQVK	HexNAc T13 13.72%
25.0	3519.6273	0.4014	GRSFYSEMLLVYCLQSLNSSGPTR	
25.0	3519.6273	0.4014	GRSFYSEMLLVYCLQSLNSSGPTR	
24.0	3519.7930	0.2356	VLSTEEIEALLKVVETERVAAEAEEAASK	
24.0	3519.7930	0.2356	VLSTEEIEALLKVVETERVAAEAEEAASK	
24.0	3519.7930	0.2356	VLSTEEIEALLKVVETERVAAEAEEAASK	
24.0	3519.7930	0.2356	VLSTEEIEALLKVVETERVAAEAEEAASK	

Mascot: http://www.matrixscience.com/

Mascot Search Results

Peptide View

MS/MS Fragmentation of **HGGTTRTADAIRYATK**
Found in **G5EGU7** in **UP1940_C_elegans**, VWFA domain-containing protein OS=Caenorhabditis elegans OX=6239 GN=C29A12.6 PE=4 SV=1

Match to Query 2435: 2125.062642 from(709.361490,3+) intensity(172035.09) scans(34748) rawscans(sn34748) rtinseconds(13085.666) index(205)
Title: 206: Scan 34748 (rt=13085.7) [D:\Adult_N2-02.raw]
Data file Adult_N2-02.temp.mgf

observedHGy15L1Gy14L2Ty13L3Ty12L4Ry11L5HETy10L6Ay9L7Dy8L8Ay7L9Iy6L10Ry5L11Yy4L12Ay3L13HETy2L14K y1L15y(8)-203937.4638 | 49.5 ppmy(9)-2031008.5017 | 45.2 ppmy(6)-203751.4018 | 59.0 ppmy(7)-203822.4363 | 57.0 ppmy(5)-203638.3185 | 68.1 ppmZoom...6007008009001000110 0m/z020406080100% of base peak0500100015002000 2500ion current

to

Monoisotopic mass of neutral peptide Mr(calc): 2124.0447
Fixed modifications: Carbamidomethyl (C) (apply to specified residues or termini only)
Variable modifications:
T7 : HexNAc (ST), with neutral losses 203.0794(shown in table), 0.0000
T15 : HexNAc (ST), with neutral losses 203.0794(shown in table), 0.0000
Ions Score: 34 **Expect:** 0.038
Peak matches: 7/288 fragment ions using 4 most intense peaks
Annotated fragments: 8/288 (help)

#	a	a++	a*	a*++	b	b++	b*	b*++	Seq.	y	y++	y*	y*++	#
1	110.0713	55.5393			138.0662	69.5367			H					16
2	167.0927	84.0500			195.0877	98.0475			G	1581.8343	791.4208	1564.8078	782.9075	15
3	224.1142	112.5607			252.1091	126.5582			G	1524.8129	762.9101	1507.7863	754.3968	14
4	325.1619	163.0846			353.1568	177.0820			T	1467.7914	734.3993	1450.7649	725.8861	13
5	426.2096	213.6084			454.2045	227.6059			T	1366.7437	683.8755	1349.7172	675.3622	12
6	582.3107	291.6590	565.2841	283.1457	610.3056	305.6564	593.2790	297.1432	R	1265.6961	633.3517	1248.6695	624.8384	11
7	683.3583	342.1828	666.3318	333.6695	711.3533	356.1803	694.3267	347.6670	T	1109.5950	555.3011	1092.5684	546.7878	10
8	754.3955	377.7014	737.3689	369.1881	782.3904	391.6988	765.3638	383.1856	A	***1008.5473***	504.7773	991.5207	496.2640	9
9	869.4224	435.2148	852.3959	426.7016	897.4173	449.2123	880.3908	440.6990	D	***937.5102***	469.2587	920.4836	460.7454	8
10	940.4595	470.7334	923.4330	462.2201	968.4544	484.7309	951.4279	476.2176	A	822.4832	411.7452	805.4567	403.2320	7
11	1053.5436	527.2754	1036.5170	518.7622	1081.5385	541.2729	1064.5119	532.7596	I	***751.4461***	376.2267	734.4196	367.7134	6
12	1209.6447	605.3260	1192.6181	596.8127	1237.6396	619.3234	1220.6131	610.8102	R	***638.3620***	319.6847	621.3355	311.1714	5
13	1372.7080	686.8577	1355.6815	678.3444	1400.7029	700.8551	1383.6764	692.3418	Y	482.2609	241.6341	465.2344	233.1208	4
14	1443.7451	722.3762	1426.7186	713.8629	1471.7401	736.3737	1454.7135	727.8604	A	319.1976	160.1024	302.1710	151.5892	3
15	1544.7928	772.9000	1527.7663	764.3868	1572.7877	786.8975	1555.7612	778.3842	T	248.1605	124.5839	231.1339	116.0706	2
16									K	147.1128	74.0600	130.0863	65.5468	1

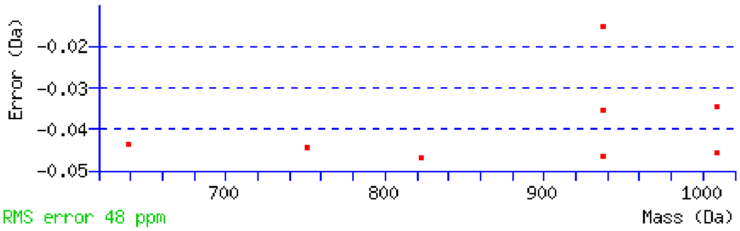

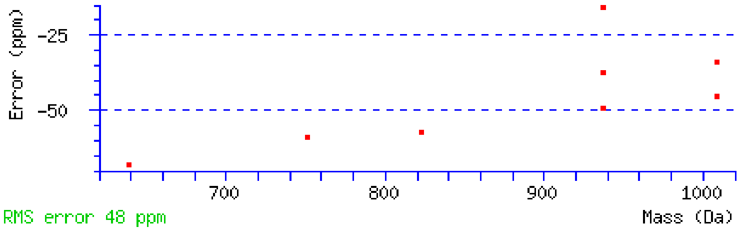

NCBI **BLAST** search of HGGTTRTADAIRYATK

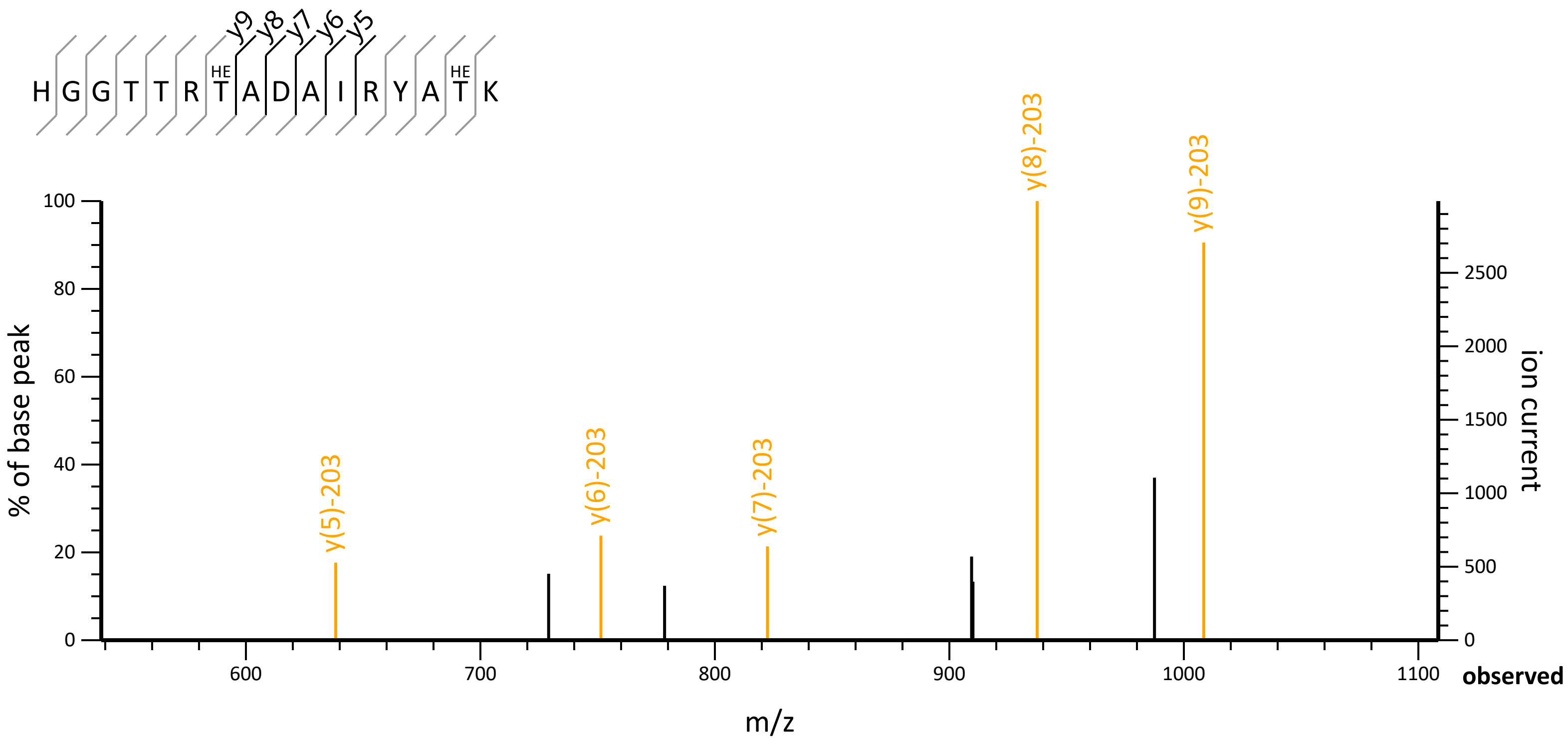

H G G T T R T A D A I R Y A T K
HE
HE
y9
y8
y7
y6
y5
y(5)-203
y(6)-203
y(7)-203
y(8)-203
y(9)-203
% of base peak
ion current
m/z
observed
0
20
40
60
80
100
500
1000
1500
2000
2500
600
700
800
900
1000
1100

(Parameters: blastp, nr protein database, expect=20000, no filter, PAM30)
Other BLAST web gateways

All matches to this query

Score	Mr(calc)	Delta	Sequence	Site Analysis
33.6	2124.0447	1.0179	HGGTTRTADAIRYATK	HexNAc T7, T15 16.67%
33.6	2124.0447	1.0179	HGGTTRTADAIRYATK	HexNAc T5, T15 16.67%
33.6	2124.0447	1.0179	HGGTTRTADAIRYATK	HexNAc T5, T7 16.67%
33.6	2124.0447	1.0179	HGGTTRTADAIRYATK	HexNAc T4, T15 16.67%
33.6	2124.0447	1.0179	HGGTTRTADAIRYATK	HexNAc T4, T7 16.67%
33.6	2124.0447	1.0179	HGGTTRTADAIRYATK	HexNAc T4, T5 16.67%
17.3	2125.0997	-0.0370	FGRYAALSLGVVYGFFR	
16.2	2125.0296	0.0330	GGTITTYKDAHNMRVMK	
16.2	2125.0296	0.0330	GGTITTYKDAHNMRVMK	
16.2	2125.0296	0.0330	GGTITTYKDAHNMRVMK	

Mascot Search Results

Peptide View

MS/MS Fragmentation of **HGGTTRTADAIRYATK**
Found in **G5EFA0** in **UP1940_C_elegans**, VWFA domain-containing protein OS=Caenorhabditis elegans OX=6239 GN=C29A12.6 PE=4 SV=1

Match to Query 2193: 2125.061592 from(709.361140,3+) intensity(112150.69) scans(34471) rawscans(sn34471) rtinseconds(13079.305) index(124)
Title: 125: Scan 34471 (rt=13079.3) [D:\L1aex3-03.raw]
Data file L1aex3-03.temp.mgf

observedHGy15a/b1Gy14a/b2Ty13a/b3HETy12a/b4Ry11a/b5HETy10a/b6Ay9a/b7Dy8a/b8Ay7a/b9Iy6a/b10Ry5a/b11Yy4a/b12Ay3a/b13Ty2a/b14Ky1a/b15y(8)937.4657 | 47.4 ppmy(9)1008.5009 | 46.0 ppma(9)++638.3082 | -22.0 ppmy(6)751.4005 | 60.7 ppmb(12)++822.4291 | -32.0 ppmy*(12)++878.4852 | -49.6 ppmZoom...600800100012001400m/z020406080100% of base peak050010001500ion current

to

Monoisotopic mass of neutral peptide Mr(calc): 2124.0447
Fixed modifications: Carbamidomethyl (C) (apply to specified residues or termini only)
Variable modifications:
T5 : HexNAc (ST), with neutral losses 0.0000(shown in table), 203.0794
T7 : HexNAc (ST), with neutral losses 0.0000(shown in table), 203.0794
Ions Score: 30 **Expect:** 0.013
Peak matches: 9/268 fragment ions using 10 most intense peaks
Annotated fragments: 9/268 (help)

#	a	a++	a*	a*++	b	b++	b*	b*++	Seq.	y	y++	y*	y*++	#
1	110.0713	55.5393			138.0662	69.5367			H					16
2	167.0927	84.0500			195.0877	98.0475			G	1987.9931	994.5002	1970.9665	985.9869	15
3	224.1142	112.5607			252.1091	126.5582			G	1930.9716	965.9895	1913.9451	957.4762	14
4	325.1619	163.0846			353.1568	177.0820			T	1873.9502	**937.4787**	1856.9236	928.9654	13
5	629.2889	315.1481			657.2838	329.1456			T	1772.9025	886.9549	1755.8759	878.4416	12
6	785.3900	393.1987	768.3635	384.6854	813.3850	407.1961	796.3584	398.6828	R	1468.7754	734.8914	1451.7489	726.3781	11
7	1089.5171	545.2622	1072.4905	536.7489	1117.5120	559.2596	1100.4855	550.7464	T	1312.6743	656.8408	1295.6478	648.3275	10
8	1160.5542	580.7807	1143.5277	572.2675	1188.5491	594.7782	1171.5226	586.2649	A	***1008.5473***	504.7773	991.5207	496.2640	9
9	1275.5812	**638.2942**	1258.5546	629.7809	1303.5761	652.2917	1286.5495	643.7784	D	***937.5102***	469.2587	920.4836	460.7454	8
10	1346.6183	673.8128	1329.5917	665.2995	1374.6132	687.8102	1357.5866	679.2970	A	***822.4832***	411.7452	805.4567	403.2320	7
11	1459.7023	730.3548	1442.6758	721.8415	1487.6972	744.3523	1470.6707	735.8390	I	***751.4461***	376.2267	734.4196	367.7134	6
12	1615.8034	808.4054	1598.7769	799.8921	1643.7984	**822.4028**	1626.7718	813.8895	R	***638.3620***	319.6847	621.3355	311.1714	5
13	1778.8668	889.9370	1761.8402	881.4237	1806.8617	903.9345	1789.8351	895.4212	Y	482.2609	241.6341	465.2344	233.1208	4
14	1849.9039	925.4556	1832.8773	916.9423	1877.8988	939.4530	1860.8722	930.9398	A	319.1976	160.1024	302.1710	151.5892	3
15	1950.9516	975.9794	1933.9250	967.4661	1978.9465	989.9769	1961.9199	981.4636	T	248.1605	124.5839	231.1339	116.0706	2
16									K	147.1128	74.0600	130.0863	65.5468	1

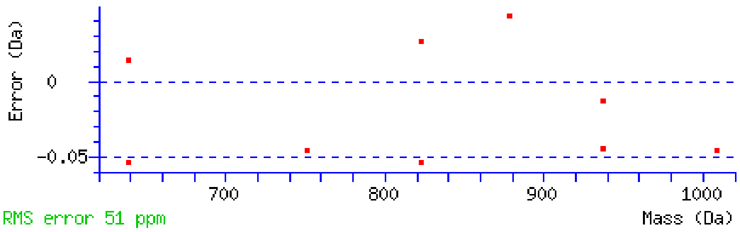

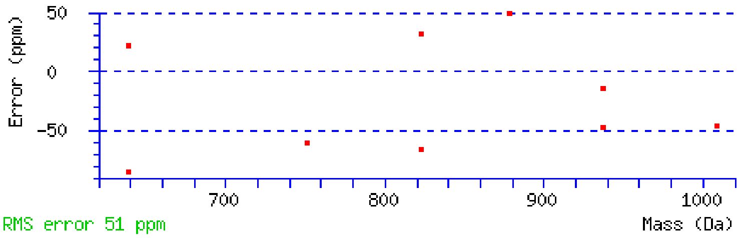

NCBI **BLAST** search of HGGTTRTADAIRYATK

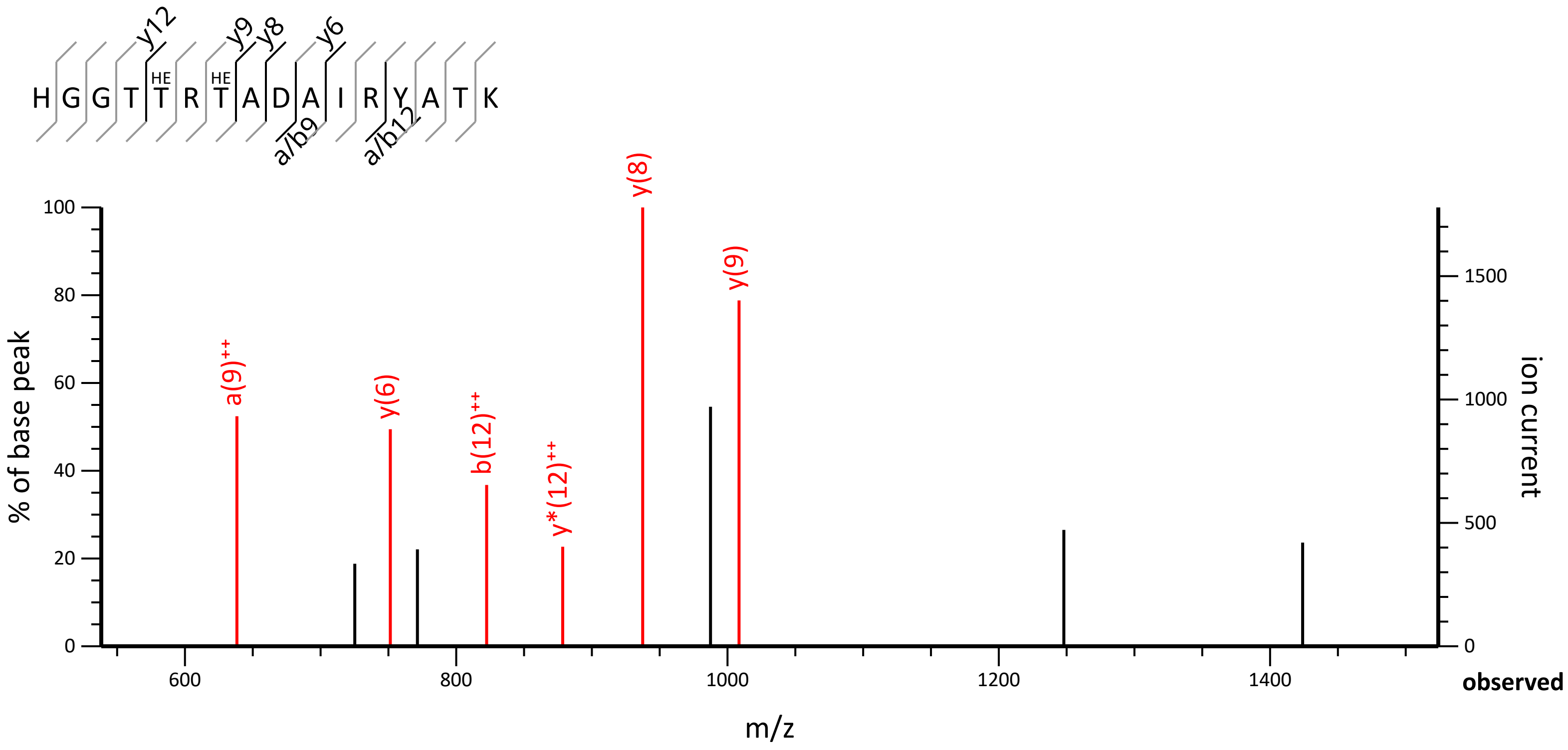

H G G T T R T A D A I R Y A T K
HE
HE
y12
y9
y8
y6
a/b9
a/b12
% of base peak
ion current
m/z
observed
a(9)++
y(6)
b(12)++
y*(12)++
y(8)
y(9)
0
20
40
60
80
100
500
1000
1500
600
800
1000
1200
1400

(Parameters: blastp, nr protein database, expect=20000, no filter, PAM30)
Other BLAST web gateways

All matches to this query

Score	Mr(calc)	Delta	Sequence	Site Analysis
29.8	2124.0447	1.0169	HGGTTRTADAIRYATK	HexNAc T5, T7 28.90%
26.7	2124.0447	1.0169	HGGTTRTADAIRYATK	HexNAc T7, T15 14.22%
26.7	2124.0447	1.0169	HGGTTRTADAIRYATK	HexNAc T5, T15 14.22%
26.7	2124.0447	1.0169	HGGTTRTADAIRYATK	HexNAc T4, T15 14.22%
26.7	2124.0447	1.0169	HGGTTRTADAIRYATK	HexNAc T4, T7 14.22%
26.7	2124.0447	1.0169	HGGTTRTADAIRYATK	HexNAc T4, T5 14.22%
9.0	2125.0997	-0.0381	FGRYAALSLGVVYGFFR	
7.1	2124.0362	1.0254	VVSSAVSTLFNTYK	
7.1	2124.0362	1.0254	VVSSAVSTLFNTYK	
6.4	2125.0296	0.0320	GGTITTYKDAHNMRVMK	

Mascot Search Results

Peptide View

MS/MS Fragmentation of **DELPAIRLISLEEDMTK**
Found in **PDI2_CAEEL** in **SwissProt**, Protein disulfide-isomerase 2 OS=Caenorhabditis elegans OX=6239 GN=pdi-2 PE=1 SV=1

Match to Query 7478: 2380.180162 from(1191.097357,2+) rtinseconds(14252.2569947) index(6115)
Title: Adult_aex3-04.39981.39981.2.0.dta
Data file Adult_aex3-04_HCDFT.mgf

observedDER16a1LR15a2PR14a3AR13a4IR12a5RR11a6LR10a7IR9a8HESR8a9LR7a10ER6a11ER5a12DR4a13MR3a14HET R2a15KR1a16a(13)-203++726.4827 | -101.7 ppma(14)-203++783.9526 | -38.7 ppma(7)++384.3037 | -207.0 ppma(4)++214.1344 | -99.9 ppmZoom...50010001500m/z020406080100% of base peak050010001500200025003000 3500ion current

to

Monoisotopic mass of neutral peptide Mr(calc): 2378.1774
Fixed modifications: Carboxymethyl (C) (apply to specified residues or termini only)
Variable modifications:
S10 : HexNAc (ST), with neutral losses 203.0794(shown in table), 0.0000
T16 : HexNAc (ST), with neutral losses 203.0794(shown in table), 0.0000
Ions Score: 22 **Expect:** 0.015
Peak matches: 4/284 fragment ions using 6 most intense peaks
Annotated fragments: 4/284 (help)

#	a	a++	a*	a*++	b	b++	b*	b*++	Seq.	y	y++	y*	y*++	#
1	88.0393	44.5233			116.0342	58.5207			D					17
2	217.0819	109.0446			245.0768	123.0420			E	1857.9990	929.5032	1840.9725	920.9899	16
3	330.1660	165.5866			358.1609	179.5841			L	1728.9564	864.9819	1711.9299	856.4686	15
4	427.2187	***214.1130***			455.2136	228.1105			P	1615.8724	808.4398	1598.8458	799.9265	14
5	498.2558	249.6316			526.2508	263.6290			A	1518.8196	759.9134	1501.7931	751.4002	13
6	611.3399	306.1736			639.3348	320.1710			I	1447.7825	724.3949	1430.7559	715.8816	12
7	767.4410	***384.2241***	750.4145	375.7109	795.4359	398.2216	778.4094	389.7083	R	1334.6984	667.8529	1317.6719	659.3396	11
8	880.5251	440.7662	863.4985	432.2529	908.5200	454.7636	891.4934	446.2504	L	1178.5973	589.8023	1161.5708	581.2890	10
9	993.6091	497.3082	976.5826	488.7949	1021.6041	511.3057	1004.5775	502.7924	I	1065.5133	533.2603	1048.4867	524.7470	9
10	1080.6412	540.8242	1063.6146	532.3109	1108.6361	554.8217	1091.6095	546.3084	S	952.4292	476.7182	935.4026	468.2050	8
11	1193.7252	597.3663	1176.6987	588.8530	1221.7201	611.3637	1204.6936	602.8504	L	865.3972	433.2022	848.3706	424.6889	7
12	1322.7678	661.8876	1305.7413	653.3743	1350.7627	675.8850	1333.7362	667.3717	E	752.3131	376.6602	735.2865	368.1469	6
13	1451.8104	***726.4088***	1434.7839	717.8956	1479.8053	740.4063	1462.7788	731.8930	E	623.2705	312.1389	606.2440	303.6256	5
14	1566.8374	***783.9223***	1549.8108	775.4090	1594.8323	797.9198	1577.8057	789.4065	D	494.2279	247.6176	477.2014	239.1043	4
15	1697.8778	849.4426	1680.8513	840.9293	1725.8728	863.4400	1708.8462	854.9267	M	379.2010	190.1041	362.1744	181.5908	3
16	1798.9255	899.9664	1781.8990	891.4531	1826.9204	913.9639	1809.8939	905.4506	T	248.1605	124.5839	231.1339	116.0706	2
17									K	147.1128	74.0600	130.0863	65.5468	1

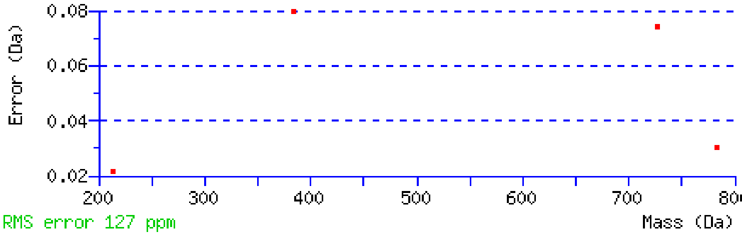

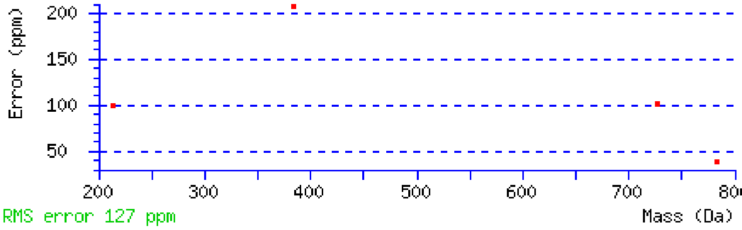

NCBI **BLAST** search of DELPAIRLISLEEDMTK
(Parameters: blastp, nr protein database, expect=20000, no filter, PAM30)

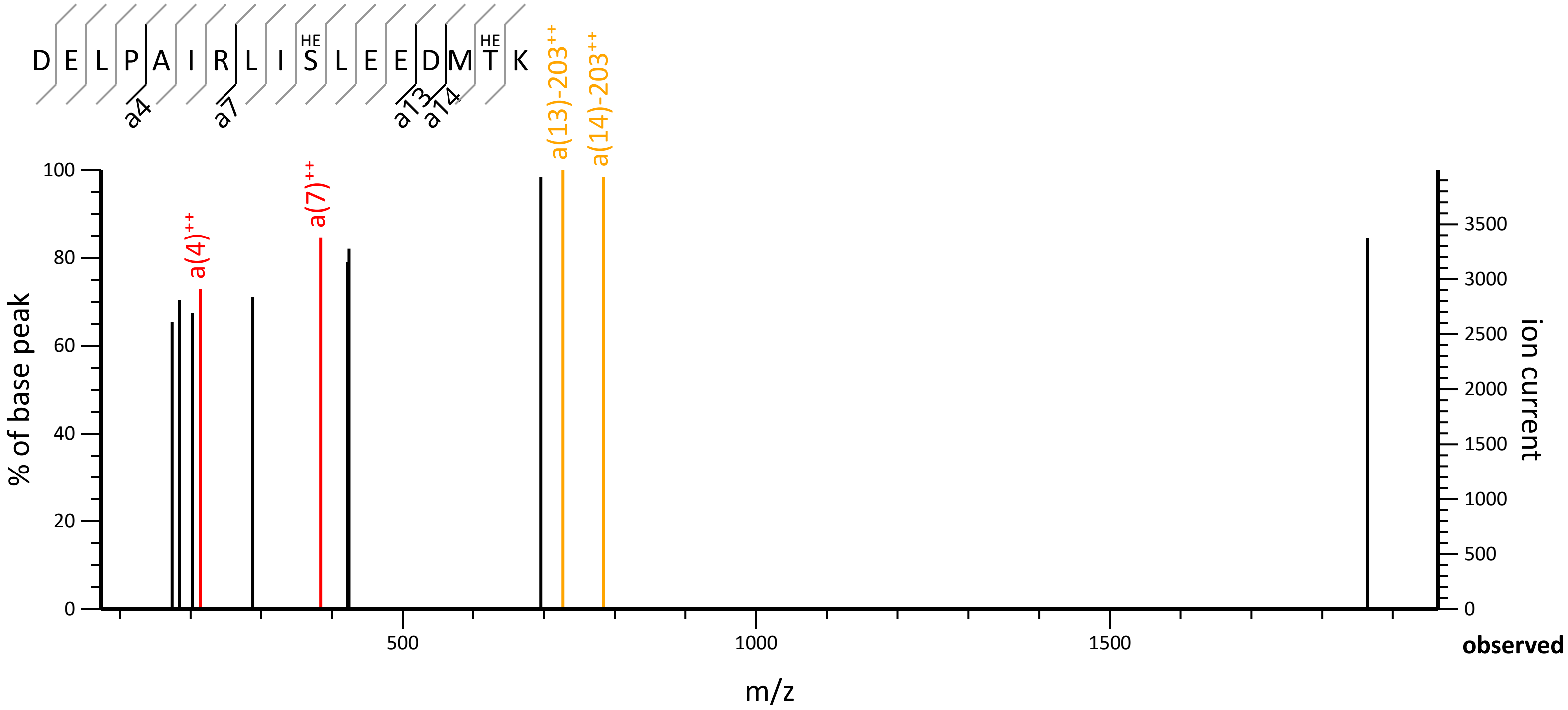

D E L P A I R L I S L E E D M T K
HE
HE
a4
a7
a13
a14
a(4)++
a(7)++
a(13)-203++
a(14)-203++
% of base peak
0
20
40
60
80
100
ion current
0
500
1000
1500
2000
2500
3000
3500
500
1000
1500
observed
m/z

Other BLAST web gateways

All matches to this query

Score	Mr(calc)	Delta	Sequence
22.2	2378.1774	2.0028	DELPAIRLISLEEDMTK
2.5	2379.1926	0.9875	YTCGNRKVIPNMPDLILR
0.0	2380.1456	0.0345	METIWIPHLHTALAYMHER
0.0	2378.2104	1.9697	GSLEKLISESYKFIR

Mascot: http://www.matrixscience.com/
